# Diet and Depression During Peri- and Post-Menopause: A Scoping Review

**DOI:** 10.3390/nu17172846

**Published:** 2025-08-31

**Authors:** Alexandra M. Bodnaruc, Miryam Duquet, Denis Prud’homme, Isabelle Giroux

**Affiliations:** 1 School of Nutrition Sciences, Faculty of Health Sciences, University of Ottawa, Ottawa, ON K1S 5S9, Canada; mduqu016@uottawa.ca (M.D.); igiroux@uottawa.ca (I.G.); 2 School of Human Kinetics, Faculty of Health Sciences, University of Ottawa, Ottawa, ON K1S 5S9, Canada; denis.prudhomme@umoncton.ca; 3 Institut du Savoir Monfort, Ottawa, ON K1K 0T2, Canada; 4 Université de Moncton, Moncton, NB E1A 3E9, Canada

**Keywords:** nutrition, dietary patterns, foods, nutrients, depression, depressive disorder, peri-menopause, post-menopause, women, scoping review

## Abstract

**Background/Objectives**: While the prevalence of depression increases during the peri- and post-menopausal periods, the potential of diet as both a modifiable risk factor and complementary treatment option has received limited research attention in this population. To address this gap, we conducted a scoping review aiming to map and synthesize the existing literature on diet and depression in peri- and post-menopause. **Methods**: Studies were identified through Medline, EMBASE, PsycINFO, CENTRAL, Web of Science, and Scopus. After deduplication in Covidence, two reviewers independently screened titles, abstracts, and full texts using predefined eligibility criteria. Data were extracted using standardized forms and presented in tables and figures. Methodological quality was assessed using the Cochrane RoB-2 for intervention studies and NHLBI tools for observational studies. **Results**: Thirty-eight studies met the inclusion criteria, including 29 observational and 9 interventional studies. Dietary patterns showed the most consistent associations with depressive symptoms, whereas findings for foods, nutrients, and other food components were inconsistent. Most observational studies had a moderate to high risk of bias, while over half of experimental studies were rated as low risk. **Conclusions**: Although limited by volume and poor methodological quality, existing evidence suggests that healthy diets may be protective against depressive symptoms in peri- and post-menopausal women, while unhealthy diets may increase risk. High-quality cohort studies and clinical trials are needed to guide future research and inform professionals working at the intersection of nutrition, psychiatry, and women’s health. **Protocol registration**: osf.io/b89r6.

## 1. Introduction

Depression is a pervasive mental health disorder affecting over 350 million individuals globally, making it the second leading cause of disability worldwide [1,2]. It is associated with psychiatric and cardiometabolic comorbidities [3,4,5], functional impairments [6,7,8,9], reduced quality of life [10,11,12,13], and substantial societal costs [6,7]. Starting in adolescence, women are approximately twice as likely as men to experience depression, a disparity that is consistently observed across countries and cultures [8]. This disparity is further amplified during life stages marked by significant hormonal changes, such as puberty, pregnancy, and the menopausal transition, which are often described as windows of vulnerability [9]. Despite being historically understudied, the menopausal transition is associated with a 2- to 5-fold increased risk of developing major depression or clinically significant depressive symptoms compared to the pre-menopausal period [14,15,16,17,18,19,20,21], even in women without a prior history of depression [16,17,18,19,20,21]. Contributing factors include hormonal fluctuations, particularly declining estrogen levels [17,18,22], as well as psychosocial stress, comorbid health conditions, and age-related changes such as sleep disturbances, weight gain, and alterations in body fat distribution [17,19]. Peri- and post-menopausal women often exhibit a distinct symptom profile characterized by rapid mood changes, irritability, paranoia, and pronounced fatigue, differing from the persistent low mood more typically observed in younger adults [23,24]. They have also shown poorer responses to standard antidepressant treatments compared to pre-menopausal women [23,25,26,27,28]. This combination of increased prevalence, atypical symptomatology, and suboptimal treatment response in midlife women underscores the need for targeted research and interventions.

In recent years, growing attention has turned to modifiable lifestyle factors as potential levers for preventing or managing depression. Among these, diet has emerged as a particularly promising target, fueling the emerging field of nutritional psychiatry. This field explores how dietary patterns and nutrient intake influence mental health, with mounting evidence associating high-quality diets, such as the Mediterranean diet and anti-inflammatory diets, with a reduced depression risk [29,30,31,32,33], and low-quality diets, characterized by high intakes of refined sugars and ultra-processed foods, with an increased depression risk [34,35,36,37]. Biological mechanisms proposed to explain these associations include inflammatory pathways [38,39,40], oxidative stress [41,42,43], hypothalamic–pituitary–adrenal axis (HPA) function [44,45], neurogenesis [46,47], and gut microbiota composition [48,49]. Chronic low-grade inflammation, in particular, is thought to mediate the association between poor diet and depression [50,51].

Despite the biological plausibility and public health significance of diet as a modifiable risk factor for depression, most research in the field has focused on general adult populations, with limited attention paid to peri- and post-menopausal women. This gap is critical, as menopause-related changes may influence not only depression risk but also how dietary factors interact with neurobiological and emotional processes. To our knowledge, no prior review has systematically mapped the existing evidence on diet and depression in this specific subgroup, despite the critical need for tailored research. We therefore conducted a scoping review aiming to map and synthesize studies examining diet-related variables and depressive symptoms in peri- and post-menopausal women. A scoping approach was selected due to the anticipated heterogeneity in study designs, dietary exposures, population characteristics, and outcome measurement tools. This methodology allowed us to systematically map available evidence and identify key gaps to inform future research. By synthesizing available findings across dietary patterns, food groups, and nutrients, this review can serve as a consolidated reference for dietitians and other healthcare providers aiming to incorporate mental health considerations into dietary assessment and counselling for midlife women.

## 2. Materials and Methods

This scoping review is reported in accordance with the Preferred Reporting Items for Systematic Reviews and Meta-Analyses extension for Scoping Reviews (PRISMA-ScR) (see Supplementary Table S1) [52]. The methods used for this scoping review were informed by Arksey & O’Malley’s (2007) framework [53] as well as Peters et al.’s (2015) guidelines for conducting scoping reviews [54]. The initial protocol was prospectively registered on the Open Science Framework (https://osf.io/b89r6/). The detailed final protocol has also been published as a protocol paper [55].

### 2.1. Deviations from the Intended Protocol

The only deviation from the published protocol [55] is that one additional database, namely the Cochrane Central Register of Controlled Trials (CENTRAL), was searched. All other steps, including study selection and data extraction, were followed as described in the published protocol [55]. Supplementary Tables S2–S6 correspond to the final search strategies used.

### 2.2. Step 1–Identifying the Research Questions

This scoping review aimed to answer the following question: “What evidence is currently available on diet-related variables and depression in peri- and post-menopausal women?”. The sub-questions were as follows:

1. What are the characteristics of the available evidence on diet-related variables and depression in peri- and post-menopausal women?
2. What are the main findings of the available evidence on diet-related variables and depression in peri- and post-menopausal women?
3. What are the main research gaps on the topic of diet-related variables and depression in peri- and post-menopausal women?

### 2.3. Step 2–Identifying the Relevant Studies

Studies examining the associations between diet-related variables and depression in peri- and post-menopausal women were systematically retrieved via Medline, EMBASE, PsycINFO, CENTRAL, Web of Science, and Scopus. The search was conducted from the inception of each database to 15 November 2024. Languages were restricted to English and French. A backward citation tracking of all included articles was conducted to identify any other pertinent articles. The search strategies for all databases are available in Supplementary Tables S2–S6 and the published protocol [55].

### 2.4. Step 3–Study Selection

To be eligible for inclusion in this review, studies were required to meet the criteria described below regarding the types of participants, exposures, interventions, comparators, outcomes, and study designs.

#### 2.4.1. Type of Participants

Studies including (1) healthy peri- and post-menopausal women or (2) peri- and post-menopausal women with diagnoses of primary major or persistent depressive disorder prior to enrollment in the study were considered eligible for this review. No restrictions were applied as to participants’ age or race. Studies were excluded if they focused exclusively on women with chronic health conditions other than those specified, women undergoing sex hormone replacement therapy, or women who had undergone hysterectomies.

#### 2.4.2. Type of Exposures and Interventions

The exposures and interventions of the included studies could include a wide range of diet-related variables. Diet-related variables were only excluded if they were considered unusual, linked to an underlying health condition or surgical procedure, or were unlikely to be found in their unaltered forms in foods. As such, while studies assessing supplements of macronutrients, vitamins, minerals, and phytonutrients (e.g., phenolic compounds, nondigestible carbohydrates) were considered eligible, those assessing herbal supplements (e.g., Ginkgo biloba, ginseng, St. John’s Wort) or any pharmaceutical agents (e.g., semaglutide, naltrexone-bupropion, etc.) aimed at modifying eating behaviors, food intake, or nutrient metabolism were excluded. No restrictions were applied as to dietary intake assessment methods in observational studies nor intervention duration in experimental studies.

#### 2.4.3. Type of Comparators

Experimental studies were considered eligible if the dietary intervention of interest was compared to (i) a placebo, (ii) another dietary intervention, or (iii) no intervention.

#### 2.4.4. Type of Outcomes

Outcomes were limited to unipolar major and persistent depressive disorder with or without current treatment, as well as to depressive symptoms. No restrictions were applied as to the methods and tools used to assess depression and depressive symptoms. Studies focusing exclusively on depressive symptoms as part of the symptomatology of another physical (e.g., hypothyroid, anemia, cardiometabolic disorders, etc.) or mental health disorders (e.g., schizophrenia, eating disorders, personality disorders, declined cognitive functions, etc.) were excluded.

#### 2.4.5. Type of Study Designs

Primary experimental (i.e., randomized controlled parallel and crossover trials with individual and cluster randomization), quasi-experimental (e.g., non-equivalent groups designs (NEGDs), difference-in-differences designs, etc.), and observational (e.g., cohort, case-control, and cross-sectional studies) studies were considered eligible. Preclinical trials, case studies, and case series were excluded.

#### 2.4.6. Selection of Studies

All records identified through the database search were imported into Covidence (Covidence, Veritas Health Innovation, Melbourne, Australia), a web-based collaboration software platform that streamlines the production of literature reviews. Duplicates were removed and the remaining records were screened against the title and abstract independently by two authors (A.M.B. and M.D.). At this stage, articles were only excluded if it was clearly determined by the title or abstract that they did not meet the inclusion criteria. Articles deemed eligible based on the title and abstract underwent full-text reviewing by the same two assessors. Prior to formal screening, a pilot calibration exercise was conducted on a sample of 50 records to ensure consistency in the application of eligibility criteria. During both title/abstract and full-text screening, discrepancies between the two independent reviewers (A.M.B. and M.D.) were resolved through discussion. Although a third reviewer (I.G.) was available for adjudication, their input was not needed.

### 2.5. Step 4–Charting the Data

One author (A.M.B.) extracted data from papers of all eligible studies. The following data was extracted: (i) authors, (ii) year of publication, (iii) protocol registration number (where applicable), (iv) protocol publication reference (where applicable), (v) study location, (vi) study design, (vii) study duration (where applicable), (viii) participant recruitment type, (ix) type of randomization (where applicable), (x) number of participants, (xi) age of participants, (xii) menopause stage(s), (xiii) name(s) of dietary variable(s), (xiv) tools used to assess dietary variables, (xv) types of variables, (xvi) outcome name, (xvii) tools used to assess depression or depressive symptoms, (xviii) quantitative results and type of statistical analysis, and (xix) adjustment variables for statistical analysis (where applicable).

### 2.6. Step 5–Collating, Summarizing, and Reporting Results

The main characteristics and findings of the included studies were summarized narratively, stratified by type of nutritional exposure or intervention, and presented in 3 tables. Research gaps are highlighted and discussed in the Discussion section of the manuscript.

### 2.7. Step 6–Methodological Quality Appraisal

The risk of bias in randomized trials, cohort and cross-sectional studies, and case-control studies was assessed using the revised Cochrane risk-of-bias (RoB-2) tool [56], the National Heart, Lung, and Blood Institute (NHLBI) Quality Assessment Tool for Observational Cohort and Cross-Sectional Studies [57], and the NHLBI Quality Assessment Tool for Case-Control studies [58], respectively.

The Cochrane RoB-2 tool assesses 5 bias domains known to affect the results of randomized trials, namely, (i) bias arising from the randomization process, (ii) bias due to deviations from intended interventions, (iii) bias due to missing outcome data, (iv) bias in the measurement of the outcomes, and (v) bias in the selection of the reported results. Each of these domains contains guiding questions, answered with “yes”, “probably yes”, “no”, “probably no”, or “no information”. Using the judgements reached for each domain, the studies themselves were rated as follows:

1. Being at low risk of bias when all domains were rated as such;
2. Raising some concerns when at least one domain was rated as such, but no domain was rated as being at high risk of bias;
3. Being at high risk of bias when at least one domain was rated as such, or when multiple domains were rated as raising some concerns.

The NHLBI Quality Assessment Tool for Observational Cohort and Cross-Sectional Studies and the NHLBI Quality Assessment Tool for Case-Control Studies consist of 14 and 12 items, respectively, assessing common sources of bias in observational studies, namely, (i) bias from participants’ recruitment, selection methods, or sample size; (ii) bias in the measurement of the exposures; (iii) bias in the measurement of the outcomes; (iv) bias due to the handling of potential confounders; and (v) bias in the selection of the reported results. Each item included in the NHLBI Quality Assessment Tools was answered by “yes”, “no”, or “no information”. Cohort and cross-sectional studies were considered to be at “low risk of bias” when the answer to ≥13 items was “yes”; at “moderate risk of bias” when the answer to 10, 11, or 12 items was “yes”; and at “high risk of bias” when the answer to <10 items was “yes”. Case-control studies were considered to be at “low risk of bias” when the answer to ≥11 items was “yes”; at “moderate risk of bias” when the answer to 8, 9, or 10 items was “yes”; and at “high risk of bias” when the answer to <8 items was “yes”.

## 3. Results

**Figure 1:**
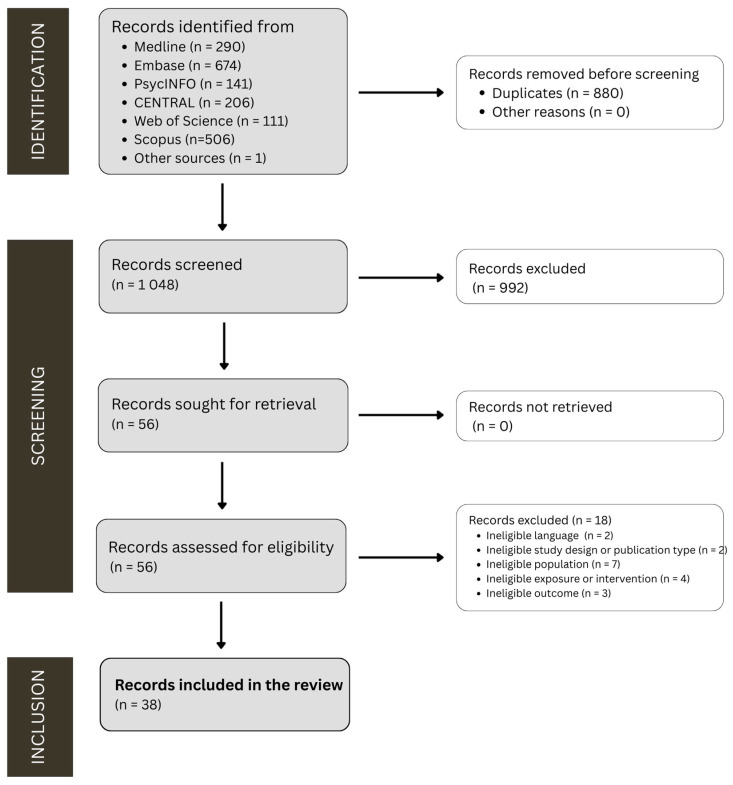
PRISMA flow diagram of the study identification and selection process.

Figure 1 presents the study selection process. Of 1929 records, 880 duplicates were removed, leaving 1048 unique records. From the total of 1048 unique records, 992 were eliminated based on title and abstract, leaving 56 records to be retrieved for full-text assessment. Of these papers, 18 were excluded: 2 due to language [59,60], 2 due to publication type [61,62], 7 due to the study population [63,64,65,66,67,68,69], 4 due to the study exposure or intervention [70,71,72,73], and 3 due to study outcome [74,75,76] (see Supplementary Table S7 for further detail on exclusion reasons), leaving 38 papers to be included in this review. Of these papers, 10 reported results from the Studies of Women’s Health Across the Nation (SWAN) [77,78,79,80,81,82,83,84,85,86] and 5 from the Women’s Health Initiative (WHI) studies [87,88,89,90,91].

### 3.1. Study Characteristics

#### 3.1.1. Type of Participants

The sample sizes of included studies ranged from 20 [92] to 81,189 [87] participants. Women were part of one of the following menopausal status groups: post-menopausal women (*n* = 21, 55.2%) [87,88,89,90,91,93,94,95,96,97,98,99,100,101,102,103,104,105,106,107,108], peri-menopausal women (*n* = 6, 15.8%) [77,79,80,81,82,84], peri- and post-menopausal women (*n* = 6, 15.8%) [92,109,110,111,112,113], or pre- and post-menopausal women (*n* = 5, 13.2%) [78,83,84,85,86].

All studies that included post-menopausal women defined post-menopause as ≥12 months of amenorrhea, based on self-reported time since last menses [87,88,89,90,91,92,93,94,95,96,97,98,99,100,101,102,103,104,105,106,107,108,109,110,111,112,113]. Most studies that included peri-menopausal women defined peri-menopause as menstrual irregularities or an absence of menses for less than 12 months, accompanied by the presence of peri-menopausal symptoms [78,83,84,85,86,92,109,110,112,113]. One study determined peri-menopausal status solely on the basis of age, while the SWAN cohort study specifically focused on the early peri-menopausal stage, defined as menstrual bleeding in the past 3 months accompanied by changes in cycle regularity [78,83,84,85,86].

#### 3.1.2. Type of Exposures, Interventions, and Comparators

Most studies focused on nutrients and other food components (*n* = 30, 78.9%, [77,78,79,80,81,82,83,84,85,86,87,88,89,92,95,96,99,101,102,103,104,105,106,107,109,110,112,113,114]), followed by dietary patterns (*n* = 7, 18.4%, [88,91,93,94,97,98,108]), and foods and food groups (*n* = 2, 5.3%, [100,111]). One study examined both nutrients and dietary patterns [88], and 7 studies assessed multiple nutrients (23.3%, [99,101,102,103,105,109,110])

Dietary patterns included whole-diet interventions in RCTs (*n* = 2, [91,108]), and dietary patterns defined *a priori* (*n* = 2, [88,94]) or *a posteriori* (*n* = 3, [93,97,98]) in observational studies (*n* = 2, [91,108]). Whole-diet interventions included the low-fat diet (*n* = 2, [91,108]) and the Dietary Approach to Stop Hypertension (DASH) (*n* = 1, [108]). *A priori* indices were the Dietary Inflammatory Index (DII) (*n* = 1, [94]) and the glycemic index (*n* = 1, [87]). Dietary patterns defined *a posteriori* included the Dietary Total Antioxidant Capacity (DTAC) (*n* = 1, [93]), healthy dietary patterns (i.e., “healthy” and “whole-plant food” diets) (*n* = 2, [97]), and highly processed dietary patterns (i.e., “processed food”, “sweets”, and “traditional Tianjin” diet) (*n* = 2, [97]).

Food and food groups included legumes (*n* = 2, [110,111]), vegetables (*n* = 1, [110]), fruit (*n* = 1, [110]), milk and plain yogurt (*n* = 1, [110]), ultra-processed foods (*n* = 1, [110]), sweet foods (*n* = 1, [110]), sugar-sweetened beverages (*n* = 1, [110]), and saffron (*n* = 1, [106]).

**Figure 2:**
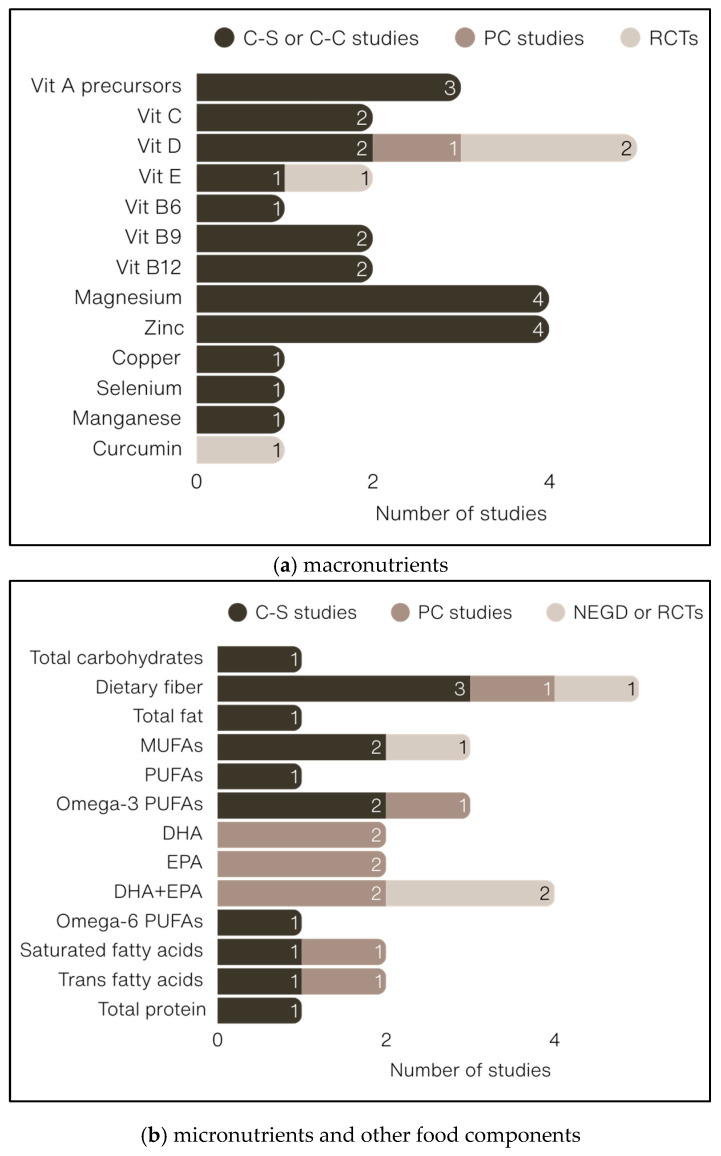
Number of studies and design types focusing on (**a**) macronutrients and (**b**) micronutrients and other food components. C-C: case-control; C-S: cross-sectional; DHA: docosahexaenoic acid; EPA: eicosapentaenoic acid; MUFA(s): monounsaturated fatty acid(s); NEGD: Non-Equivalent Groups Design; PC: prospective cohort; PUFA(s): polyunsaturated fatty acid(s); RCTs: randomized controlled trials; Vit; vitamin.

As illustrated in Figure 2, nutrients included the following:

- dietary fiber [81,88,96,110,113]; vitamin D_3_ [87,90,107,110,114]) (*n* = 5 each);
- eicosapentaenoic acid (EPA) + docosahexaenoic acid (DHA) [89,92,104,112]; magnesium [99,102,103,110]; zinc [99,102,103,110] (*n* = 4 each);
- vitamin A and vitamin A precursors (*n* = 3, [82,83,109]);
- vitamin E [105,109,110]; vitamin B_9_ [101,109,110] (*n* = 3 each);
- EPA [89,104], DHA [89,104], total omega-3 fatty acids [95,110], saturated fatty acids (SFAs) [85,110], *trans* fatty acids [86,110], vitamin B_12_ [101,110], vitamin C [84,110], copper [103,110], selenium [103,110] (*n* = 2 each);
- total carbohydrates [110], added sugar [88], total lipids [110], monounsaturated fatty acids (MUFAs) [110], total omega-6 fatty acids [110], linoleic acid [78], oleic acid [78], total proteins [110], manganese [80], and curcumin [105] (*n* = 1 each).

#### 3.1.3. Type of Outcomes

Depressive symptoms were assessed using various validated tools, namely the Center for Epidemiologic Studies Depression Scale (CES-D) (*n* = 16, [77,78,79,80,81,82,83,84,85,86,91,98,101,104,110,111]), the Beck Depression Inventory (BDI) (*n* = 4, [99,102,103,114]), the 9-item Patient Health Questionnaire (PHQ) (*n* = 3, [94,96,109]), the 8-item Burnam Scale (BS) (*n* = 3, [89]), the 22-item Depression Anxiety Stress Scale (DASS) (*n* = 2, [93]), the 21-item Hamilton Depression Rating Scale (HDRS) (*n* = 1, [106]), the Montgomery–Åsberg Depression Rating Scale (MADRS) (*n* = 1, [92]), the 20-Item Hopkins Symptoms Checklist Depression Scale (HSCL-D) (*n* = 1, [112]), the 18-item Brief Symptom Inventory (BSI) (*n* = 1, [107]), the Zung Self-Rating Depression Scale (ZSRDS) (*n* = 1, [97]), the Women’s Health Questionnaire (WHQ) (*n* = 1, [100]), the Greene Climacteric Scale (GCS) (*n* = 1, [105]), the depression subscale of the 37-item Profile of Mood State (POMS) (*n* = 1, [108]), and an unspecified scale (*n* = 1, [95]). In addition to the use of a questionnaire to identify depressive symptoms, some studies considered participants taking antidepressants as having depressive symptoms (*n* = 6, [87,88,89,90,104,111]), and one study used self-reported depression diagnosis as indicative of depressive symptoms [95].

#### 3.1.4. Type of Study Designs

**Figure 3:**
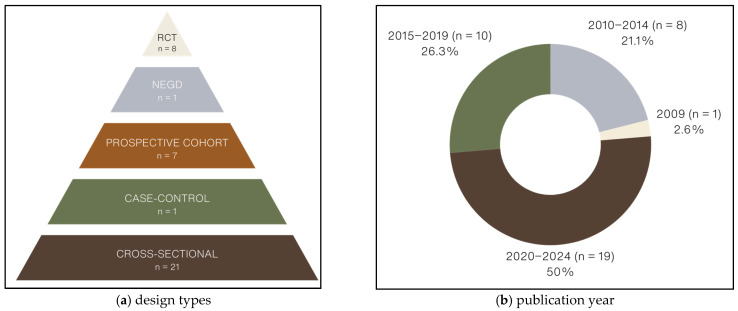
Distribution of included studies by: (**a**) design type and (**b**) publication year. NEGD: non-equivalent group design; RCT: randomized controlled trial.

As shown in Figure 3a, out of the 38 studies included in this review, 29 (76.3%, [73,77,78,79,80,81,82,83,84,85,86,87,88,89,93,94,95,96,97,98,100,101,102,103,104,109,110,111,114]) were observational, 8 (21.1%, [90,91,105,106,107,108,112,113]) were randomized-controlled trials (RCTs), and 1 (2.6%, [92]) was a quasi-experimental pretest-post-test NEGD study. Observational studies were cross-sectional (*n* = 21, 72.4%, [73,77,78,79,80,81,82,83,84,93,94,95,96,97,98,100,101,102,103,109,110,114]), prospective cohort (*n* = 7, 24.1%, [85,86,87,88,89,104,111]), or case-control (*n* = 1, 3.4%, [99]) studies.

Articles included in this review were all published between 2009 and 2024. As shown in Figure 3b, of these, 19 papers (50.0%, [73,77,78,79,80,81,82,83,84,85,86,94,95,96,103,105,109,110,113,114]) were published between 2020 and 2024, 10 papers (26.3%, [88,91,93,97,98,99,100,104,106,107]) were published between 2015 and 2019, 8 papers (21.1%, [87,89,90,92,101,102,108,111]) were published between 2010 and 2014, and 1 paper (2.6%, [112]) was published in 2009. Over half (*n* = 22, 57.9%, [73,77,78,79,80,81,82,83,84,85,86,87,88,89,90,91,92,94,96,104,107,111,112]) of included studies were conducted in North American countries, 10 studies (26.3%, [93,95,97,98,99,101,105,106,109,113]) were conducted in Asian countries, 3 studies (7.9%, [102,103,114]) were conducted in European countries, 2 studies (5.3%, [100,110]) were conducted in South American countries, and 1 study (2.6%, [108]) was conducted in Australia.

#### 3.1.5. Methodological Quality

Out of the 29 observational studies, 3 (10.3%, [87,88,89]) were at low risk of bias, 5 (17.2%, [85,86,96,104,111]) were at moderate risk of bias, while the remaining 21 (72.4%, [73,76,77,78,79,80,81,82,83,84,93,94,95,97,98,99,100,101,102,103,109,110,114]) were at high risk of bias (see Supplementary Tables S9 and S10). Of the 9 (quasi)-experimental studies, 5 (55.6%, [105,106,107,112,113]) were at low risk of bias, while the remaining 4 (44.4%, [90,91,92,108]) were at high risk of bias (see Supplementary Table S11).

### 3.2. Summary of Study Findings

**Figure 4:**
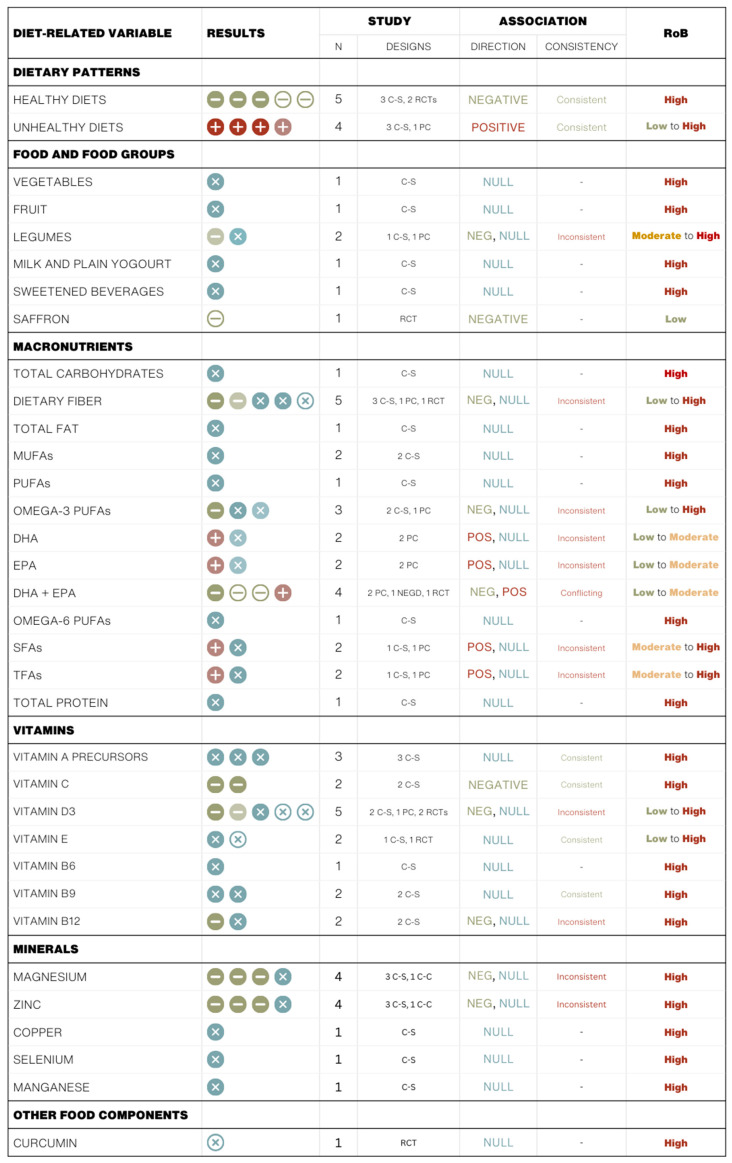
Summary of the direction, significance, and consistency of associations between dietary-related variables and depressive symptoms in peri- and post-menopausal women. Each icon represents a single study. Full dark-colored circles indicate cross-sectional (C-S) or case-control (C-C) studies, full light-colored circles indicate prospective cohort (PC) studies, and empty circles represent quasi-experimental studies (i.e., non-equivalent group design (NEGD) trial) and RCT color coding reflects the direction and statistical significance of associations: blue indicates null associations (×), green indicates statistically significant negative associations (−), and red indicates statistically significant positive associations (+).

Table 1, Table 2 and Table 3 summarize the characteristics and findings of cross-sectional and case-control studies, cohort studies, and (quasi)-experimental studies, respectively. Figure 4 provides a graphical summary of the direction and significance of associations between diet-related variables and depressive symptoms in peri- and post-menopausal women.

#### 3.2.1. Dietary Patterns

*Healthy Dietary Patterns*

Associations between healthy dietary patterns and depressive symptoms were examined in 3 cross-sectional studies [93,97,98] and 2 RCTs [91,108], all conducted in post-menopausal women. Cross-sectional studies (*n* = 222 to 953) found that higher DTAC [93], adherence to a “healthy” diet [97], and adherence to a “whole-plant food” diet [98] were associated with fewer depressive symptoms. In a 1-year open-label RCT (*n* = 48,834), a low-fat diet significantly reduced depressive symptoms compared with no intervention [91]. Similarly, a 14-week RCT (*n* = 95) found that both DASH and low-fat diets significantly reduced depressive symptoms from baseline to post-intervention, with no significant difference between groups [108].

**Table 1:** Study characteristics and findings summary from cross-sectional (*n* = 21) and case-control * (*n* = 1) studies on diet and depressive symptoms in peri- and post-menopausal women.

Authors (Year) *Country*	Population	Exposure	Outcome	Statistical Adjustments	Results	RoB
Abshirini et al. (2019) [93] *Iran*	*n* = 175 Post-MP	DTAC Method: FFQ (147 items) and PCA	Depressive symptoms Method: DASS-42	SECs; MPSs	DTAC was negatively associated with depressive symptoms (β = −0.11, *p* = 0.03).	9/15 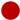
Azarmanesh et al. (2022) [94] *United States*	*n* = 2392 Post-MP	DII Method: 24 h dietary recall and DII	Depressive symptoms Method: PHQ-9	SECs; Anthropometrics; Health behaviors	DII was positively associated with depressive symptoms (Q4 vs. Q1, OR: 2.1, 95%CI: 1.1–4.3).	9/15 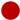
Chae et al. (2021) [95] *South Korea*	*n* = 4150 Post-MP	Omega-3 PUFA intake Method: 24 h dietary recall	Depression dx or symptoms Method: Dx or NR tool	SECs; Anthropometrics; Health behaviors; Diet	Omega-3 PUFA intake was negatively associated with depression dx or symptoms (Q5 vs. Q1, OR: 0.52, 95%CI: 0.33–0.83).	9/15 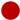
Kim et al. (2021) [96] *United States*	*n* = 2858 Post-MP	Dietary fiber intake Method: 24 h dietary recall	Depressive symptoms Method: PHQ-9	SECs; Anthropometrics; Health behaviors; Chronic diseases	Dietary fiber intake was not associated with depressive symptoms.	10/15 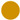
Kostecka et al. (2022) [114] *Poland*	*n* = 191 Peri-MP	Vit. D_3_ status Method: NR	Depressive symptoms Method: BDI	None reported	Vit. D3 status was not associated with depressive symptoms.	8/15 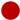
Lee et al. (2023) [109] *South Korea*	*n* = 1770 Peri/Post-MN	Vit. B_9_, A, and E serum levels Method: NR	Depressive symptoms Method: PHQ-9	SECs; Health behaviors	Vit. B9, A, and E serum levels were not associated with depressive symptoms.	9/15 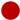
Li et al. (2020a) [77] *United States*	*n* = 1406 Peri-MN	Omega-3 PUFA intake Method: FFQ (103 items)	Depressive symptoms Method: CES-D	SECs; Anthropometrics; Health behaviors; Diet; SHs	Omega-3 PUFA intake was negatively associated with depressive symptoms (Q4 vs. Q1, OR: 0.06, 95%CI: 0.01–0.46).	9/15 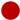
Li et al. (2020b) [78] *United States*	*n* = 2793 Pre/peri-MN	Oleic and linoleic acid intakes Method: FFQ (103 items)	Depressive symptoms Method: CES-D	SECs; MPSs; Anthropometrics; Health behaviors	Oleic (Q4 vs. Q1, OR: 2.00, 95%CI: 1.30–3.06) and linoleic (Q4 vs. Q1, OR: 1.59, 95%CI: 1.05–2.42) acid intakes were positively associated with depressive symptoms, even when adjusted for MPSs.	9/15 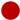
Li et al. (2020c) [79] *United States*	*n* = 1403 Peri-MN	TFA intake Method: FFQ (103 items)	Depressive symptoms Method: CES-D	SECs; Anthropometrics; Health behaviors; Diet	TFA intake was not associated with depressive symptoms.	9/15 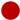
Li et al. (2020d) [80] *United States*	*n* = 1359 Peri-MN	Mn intake Method: FFQ (103 items)	Depressive symptoms Method: CES-D	SECs; Anthropometrics; Health behaviors; Diet; VMSs	Mn intake was not associated with depressive symptoms.	9/15 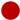
Li et al. (2020e) [81] *United States*	*n* = 1403 Peri-MN	Dietary fiber intake Method: FFQ (103 items)	Depressive symptoms Method: CES-D	SECs; Anthropometrics; Health behaviors; Diet; SHs	Dietary fiber intake was not associated with depressive symptoms.	9/15 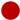
Li et al. (2021) [82] *United States*	*n* = 1400 Peri-MN	β-carotene intake Method: FFQ (103 items)	Depressive symptoms Method: CES-D	SECs; Anthropometrics; Health behaviors; Diet; SHs; VMSs	β-carotene intake was not associated with depressive symptoms.	9/15 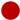
Li et al. (2022a) [83] *United States*	*n* = 3054 Pre/peri-MN	Provit. A intake Method: FFQ (103 items)	Depressive symptoms Method: CES-D	SECs; Anthropometrics; Health behaviors; Diet; SHs	Provit. A intake was not associated with depressive symptoms, even when adjusted for MPSs.	9/15 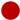
Li et al. (2022b) [84] *United States*	*n* = 3088 Pre/peri-MN	Vit. C intake Method: FFQ (103 items)	Depressive symptoms Method: CES-D	SECs; Health behaviors; Diet; Chronic diseases	Vit. C intake was negatively associated with depressive symptoms (OR: 0.70, 95%CI: 0.52–0.93), even when adjusted for MPSs.	9/15 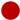
Liao et al. (2019) [97] *China*	*n* = 2051 Post-MP	Dietary patterns *a posteriori* Method: FFQ (100 items) and PCA	Depressive symptoms Method: ZSRDS	SECs; Health behaviors; Diet; Chronic diseases	The “healthy” dietary pattern was negatively associated with depressive symptoms (Q4 vs. Q1, OR: 0.57, 95%CI: 0.33–0.97). The “sweets” (Q4 vs. Q1, OR: 1.66, 95%CI: 1.03–2.71) and “traditional Tianjin” (Q4 vs. Q1, OR: 2.53, 95%CI: 1.58–4.16) dietary pattern were positively associated with depressive symptoms.	9/15 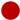
Liu et al. (2016) [98] *China*	*n* = 1125 Post-MP	Dietary patterns *a posteriori* Method: FFQ (85 items) and PCA	Depressive symptoms Method: CES-D	SECs; Health behaviors; Diet; Chronic diseases	The “whole-plant food” processed food dietary pattern was positively associated with depressive symptoms (T3 vs. T1, OR: 1.79, 95%CI: 1.18–2.72). The “processed food” dietary pattern was positively associated with depressive symptoms (T3 vs. T1, OR: 1.79, 95%CI: 1.18–2.72). The “animal food” dietary pattern was not associated with depressive symptoms.	9/15 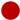
Nazari et al. (2019) * [99] *Iran*	*n* = 136 Post-MP	Mg and Zn serum levels Method: AAS	Depressive symptoms Method: BDI	NR	Zn (OR: 0.97, 95%CI: 0.96–0.99) and Mg (OR: 0.30, 95%CI: 0.15–0.61) serum levels were negatively associated with depressive symptoms.	7/15 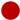
Noll et al. (2022) [100] *Brazil*	*n* = 225 Post-MP	Intake of 7 food groups Method: 24 h dietary recall	Depressive symptoms Method: WHQ	SECs; MPSs	Vegetable intake was negatively associated with depressive symptoms (T2-3 vs. T1, PR: 0.65, 95%CI: 0.43–0.98). Ultra-processed food, sweet food, sugar sweetened beverage, fruit, legume, and milk and plain yogurt intakes were not associated with depressive symptoms.	9/15 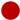
Oldra et al. (2020) [110] *Brazil*	*n* = 400 Peri/post-MP	Intake of 19 nutrients Method: 3 d food diary	Depressive symptoms Method: CES-D	NR	Dietary fiber, PUFA, Mg, Zn, vit. C, D3, and B12 intakes were negatively associated with depressive symptoms (*p* < 0.05). Carbohydrate, protein, lipid, SFA, MUFA, omega-3 and omega-6 PUFA, Se, vit. B6, and B9 intakes were not associated with depressive symptoms.	9/15 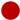
Şengül et al. (2014) [101] *Turkey*	*n* = 96 Post-MP	Serum vit. B_9_ and B_12_ levels Method: Autoanalyzer	Depressive symptoms Method: CES-D	NR	Serum vit. B9 and B12 levels did not differ between women with and without depressive symptoms.	7/15 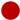
Stanisławska et al. (2014) [102] *Pomeranian region*	*n* = 171 Post-MN	Plasma Mg and Zn levels Method: AAS	Depressive symptoms Method: BDI	NR	Mg plasma levels were lower in women with mild depressive symptoms than women without (*p* < 0.05). Zn plasma levels were lower in women with moderate depressive symptoms than women without (*p* < 0.05).	7/15 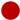
Wieder-Huszla et al. (2020) [103] *Pomeranian region*	*n* = 102 Post-MP	Mg, Zn, Cu, and Se serum levels Method: Mannovette system	Depressive symptoms Method: BDI-II	NR	Mg, Zn, Cu, and Se serum levels were not associated with depressive symptoms.	7/15 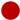

In the RoB column, orange circles indicate moderate RoB, while red circles indicate high RoB. * Case-control study. AAS: atomic absorption spectrometry; BDI: Beck’s Depression Inventory; CES-D: Centre for Epidemiologic Studies Depression Scale; CI: confidence interval; Cu: copper; d: day; DII: Dietary Inflammatory Index; DASS: Depression Anxiety Stress Scale; DTAC: dietary total antioxidant capacity; Dx: diagnosis; FFQ: Food Frequency Questionnaire; h: hour(s); MRS: Menopause Rating Scale; Mg: magnesium; Mn: manganese; MP: menopause; MPSs: menopausal symptoms; MRS: Menopause Rating Scale; MUFA(s): monounsaturated fatty acid(s); NR: non/not/none reported; OR: odds ratio; ORAC: oxygen radical absorbance capacity; PCA: principal component analysis; PHQ: Patient Health Questionnaire; PR: prevalence ratio; PUFA(s): polyunsaturated fatty acid(s); Q(1–4): quartile 1–4; Q(1–5): quintile 1–5; SFA(s): RoB: risk of bias; Se: selenium; SECs: socioeconomic Characteristics; SFA(s): saturated fatty acid(s); SHs: sex hormones; T(1–3): tertile 1–3; TFA(s): trans fatty acid(s); Vit; vitamin; VMSs: vasomotor symptoms; Vs: versus; WHQ: Women Health Questionnaire; Zn: Zinc; ZSRDS: Zung Self-Rating Depression Scale DHA: docosahexaenoic acid; EPA: eicosapentaenoic acid; MUFA(s): monounsaturated fatty acid(s); PUFA(s): polyunsaturated fatty acid(s); Vit; vitamin.

**Table 2:** Study characteristics and findings summary from prospective cohort studies (*n* = 7) on diet and depressive symptoms in peri- and post-menopausal women.

Authors (Year) *Country*	Population	Exposure	Outcome	Statistical Adjustments	Results	**RoB**
Bertone-Johnson et al. (2011) [87] *United States* **Study duration:** 3 y	*n* = 81,189 Post-MP	Vit. D_3_ intake Method: FFQ (122 items)	Depressive symptoms Method: 8-BS/AD use	SECs; Anthropometrics; Health behaviors; HT use; Diet; Chronic diseases; Solar irradiance	Compared to vit. D3 intakes < 100 IU, vit. D3 intakes ≥ 400 IU and <800 IU were associated with a lower risk of depressive symptoms (OR: 0.88, 95%CI: 0.79–0.97).	13/15 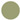
Colangelo et al. (2017) [104] *United States* **Study duration:** 3.2 y	*n* = 1616 Post-MP	DHA, EPA, and DHA+EPA intakes Method: FFQ (120 items)	Depressive symptoms Method: CES-D/AD use	SECs; Anthropometrics; Health Behaviors; Diet; Chronic diseases	DHA (Q4 vs. Q1, RR: 2.39, 95%CI: 1.45–3.39), EPA (Q4 vs. Q1, RR: 2.10, 95%CI: 1.27–3.48), and EPA+DHA (Q4 vs. Q1, RR: 2.04, 95%CI: 1.24–3.37) intakes were positively associated with depressive symptoms.	12/15 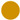
Gangwisch et al. (2015) [88] *United States* **Study duration:** 3 y	*n* = 69,954 Post-MP	Dietary glycemic index Added sugar intake Dietary fiber intake Method: FFQ (145 items)	Depressive symptoms Method: 8-BS/AD use	SECs; Anthropometrics; Health behaviors; Social support; Stressful life events; HT use; Diet; Chronic diseases	Dietary glycemic index (Q5 vs. Q1, OR: 1.22, 95%CI: 1.09–1.37) and added sugar intake (Q5 vs. Q1, OR: 1.23, 95%CI: 1.07–1.41) were positively associated with depressive symptoms. Dietary fiber intake was negatively associated with depressive symptoms (Q5 vs. Q1, OR: 0.86, 95%CI: 0.76–0.98).	13/15 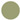
Li et al. (2010) [102,111] *United States* **Study duration:** 10.6 y	*n* = 1005 Peri/Post-MP	Weekly legume intake Method: FFQ	Severe depressed mood Method: CES-D/AD use	SECs; Anthropometrics; Health behaviors; Diet; Food Allergies; Chronic diseases	In peri-MP women, only moderate (1-2x/wk.) vs. infrequent (<1x/wk.) legume intake was associated with a lower risk of severe depressed mood (RR: 0.52, 95%CI: 0.27–1.00). In post-MP women, weekly legume consumption was not associated with severe depressed mood.	12/15 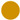
Li et al. (2020f) [85] *United States* **Study duration:** 5 y	*n* = 2376 Pre/Post-MP	SFA intake Method: FFQ (103 items)	Depressive symptoms Method: CES-D	SECs; Anthropometrics; Health behaviors; Chronic stress; AD use; Diet; VMSs; SHs	SFA intake was positively associated with depressive symptoms (Q4 vs. Q1, OR: 2.61, 95%CI: 1.15–5.93), even when adjusted for MPS.	12/15 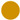
Li et al. (2020g) [86] *United States* **Study duration:** 5 y	*n* = 2376 Pre/Post-MP	TFA intake Method: FFQ (103 items)	Depressive symptoms Method: CES-D	Anthropometrics; Health behaviors; Chronic stress; AD use; Diet; SHs	TFA intake was positively associated with depressive symptoms (Q4 vs. Q1, OR: 1.64, 95%CI: 1.09–2.47), even when adjusted for MPS.	12/15 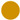
Persons et al. (2014) [89] *United States* **Study duration:** 3 y	*n* = 7066 Post-MP	Omega-3 PUFA intake DHA, EPA, DHA+EPA intakes RBC omega-3 PUFAs, DHA, and EPA Methods: FFQ (120 items; intake) NR (RBC)	Depressive symptoms Method: 8-BS/AD use	SECs; Health behaviors; HT use; Bilateral oophorectomy; Chronic diseases	Omega-3 PUFA, DHA, and EPA intakes were not associated with depressive symptoms. DHA+EPA intakes were negatively associated with depressive symptoms (T3 vs. T1, OR: 0.71, 95%CI: 0.50–0.99). RBC omega-3 PUFA, DHA, EPA, and DHA+EPA levels were not associated with depressive symptoms.	13/15 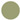

In the RoB column, green circles indicte low RoB, while orange circles indicate moderate RoB. AD: antidepressant; BS: Burnam Scale; CES-D: Centre for Epidemiologic Studies Depression Scale; CI: confidence interval; DHA: docosahexaenoic acid; EPA: eicosapentaenoic acid eicosapentaenoic; FFQ: food frequency Questionnaire; HT: hormone therapy; IU: international units; MP: menopause; MPS: menopausal status; OR: odds ratio; PUFA(s): polyunsaturated fatty acid(s); Q(1–4): quartile 1–4; RBC: red blood cell; RoB: risk of bias; RR: risk ratio; SECs: socioeconomic characteristics; SHs: sex hormones; SFA(s): saturated fatty acid(s); T(1–3): tertile; TFA(s): trans fatty acid(s); Vit(s): vitamin(s); VMSs: vasomotor symptoms; Vs: versus; Wk.: week; y: year(s).

**Table 3:** Study characteristics and findings summary from (quasi)-experimental studies (*n* = 9) on diet and depressive symptoms in peri- and post-menopausal women.

Authors (Year) *Country*	Study Design	Population	Interventions		Outcome	Results	RoB
			Experimental	Control			
Assaf et al. (2016) [91] *United States*	Open-label RCT **Duration:** 1 y *n* = 48,834	Post-MP	Low-fat diet	No intervention	Depressive symptoms Method: Modified CES-D	The low-fat diet (vs. no intervention) decreased depressive symptoms (MD: 0.07, 95%CI: 0.02–0.12).	High 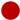
Bertone-Johnson et al. (2012) [90] *United States*	TB-RCT **Duration:** 3 y *n* = 36,282	Post-MP	Daily vit. D_3_ (400 IU) + Ca (1000 mg) supplement capsules	Placebo capsule	Depressive symptoms Method: 8-BS/AD use	No significant difference was observed between the effects of the experimental and control interventions.	High 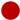
Farshbaf-Khalili et al. (2022) [105] *Iran*	TB-RCT **Duration:** 8 wks. *n* = 81	Post-MP	Experimental intervention #1: Daily curcumin (1000 mg) supplement capsules Experimental intervention #2**:** Daily vit. E (1000 mg) supplement capsules	Placebo capsule	Depressive symptoms Method: GCS	All interventions decreased depressive symptoms from pre- to post-intervention, no significant difference was observed between their individual effects.	Low 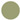
Freeman et al. (2011) [92] *United States*	Pre-Post NEGD **Duration:** 8 wks. *n* = 20	Peri/Post-MP	Daily ethyl-DHA (375 mg) + EPA (465 mg) supplement capsules	None	Depressive symptoms Method: MADRS	The intervention decreased depressive symptoms from pre- to post-intervention (MD: −12.0, SD: 8.3, *p* < 0.0001).	High 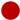
Kashani et al. (2018) [106] *Iran*	DB-RCT **Duration:** 6 wks. *n* = 56	Post-MP	Daily saffron (30 mg) supplement capsules	Placebo capsule	Depressive symptoms Method: HDRS	The experimental intervention (vs. control) significantly decreased depressive symptoms (SMD: 19.6, 95%CI: 9.00–30.28, *p* = 0.001).	High 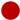
Lucas et al. (2009) [112] *Canada*	TB-RCT **Duration:** 8 wks. *n* = 120	Peri/Post-MP	Daily ethyl-DHA (150 mg) + EPA (1005 mg) supplement capsules	Placebo capsule	Depressive symptoms Method: HSCL-D-2 and HDRS-21	The experimental intervention (vs. control) decreased depressive symptoms when assessed with the HSCL-D-20 (SMD: −0.85 vs. −0.50, *p* < 0.05) and the HDRS-21 (SMD: −0.74 vs. −0.38, *p* < 0.05). These effects were, however, restricted to women without a major depressive episode at baseline (*n* = 91).	Low 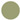
Mason et al. (2016) [107] *United States*	TB-RCT **Duration:** 1 y *n* = 218	Post-MP	Daily vit. D_3_ (2000 IU) supplement capsules	Placebo capsule	Depressive symptoms Method: BSI-18	No significant difference was observed between the effects of the experimental and control interventions.	Low 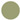
Shafie et al. (2022) [74] *Iran*	TB-RCT **Duration:** 6 wks. *n* = 60	Peri/Post-MP	Prebiotic-enriched yoghurt	Regular yoghurt placebo	Depressive symptoms Method: DASS-21	No significant difference was observed between the effects of the experimental and control interventions.	Low 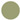
Torres et al. (2012) [108] *Australia*	Open-label RCT **Duration:** 14 wks. *n* = 95	Post-MP	Experimental intervention #1: DASH diet Experimental intervention #2: Low-fat diet	None	Depressive symptoms Method: 37-item POMS	Both the DASH diet (MD: −1.1, SEM: 0.8, *p* < 0.01) and the low-fat diet (MD: −0.6, SEM: 0.4, *p* < 0.01) decreased depressive symptoms from pre- to post-intervention, but no significant difference was observed between their individual effects.	High 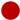

In the RoB column, green circles indicate low RoB, while red circles indicate high RoB. AD: antidepressant; BS: Burnam Scale; BSI: Brief Symptom Inventory; Ca: calcium; CES-D: Centre for Epidemiologic Studies Depression Scale; CI: confidence interval; DASH: Dietary Approach to Stop Hypertension; DASS: Depression Anxiety Stress Scale; DB-RCT: double-blind randomized controlled trial; DHA: docosahexaenoic acid; EPA: eicosapentaenoic acid; Exp.: experimental intervention; GCS: Greene Climacteric Scale; HDRS: Hamilton Depression Rating Scale; HSCL: Hopkins Symptoms Checklist Depression Scale; IU: international units; MADRS: Montgomery–Åsberg Depression Rating Scale; MD: mean difference; MP: menopause; NEGD: Non-Equivalent Groups Design; POMS: Profile of Mood State; RoB: risk of bias; RT: randomized trial; SD: standard deviation; SEM: standard error of the mean; SMD: standard mean difference; TB-RCT: triple-blind randomized controlled trial; Vit(s): vitamin(s); Wk.(s); week(s); y: year(s).

*Unhealthy Dietary Patterns*

Associations between unhealthy dietary patterns and depressive symptoms were examined in 3 cross-sectional studies [94,97,98] and 1 prospective cohort study [88], all conducted in post-menopausal women. The 3 cross-sectional studies found that higher DII scores (*n* = 393) [94], greater adherence to “sweets” and “traditional Tianjin” dietary patterns (*n* = 906) [97], and greater adherence to “processed food” and “animal food” dietary patterns (*n* = 953) [98] were associated with a higher prevalence of depressive symptoms. A prospective cohort study (*n* = 87,618; 3-year follow-up) also found a positive association between baseline dietary glycemic index and depressive symptoms at follow-up [88].

#### 3.2.2. Food and Food Groups

Associations between foods and food groups and depressive symptoms were examined in 1 cross-sectional study [100], 1 prospective cohort study [111], and 1 RCT [106]. The cross-sectional study, conducted in post-menopausal women (*n* = 213), found a negative association between vegetable intake and depressive symptoms, while intakes of fruit, legumes, milk and plain yogurt, and sugar-sweetened beverages were not significantly associated with depressive symptoms [100]. The prospective cohort study (10-year follow-up) found a U-shaped association between legume intake and severe depressed mood in peri-menopausal women (*n* = 1657), but not in post-menopausal women (*n* = 1645) [111]. Specifically, in peri-menopausal women, moderate (1-2x/wk.) legume intake was associated with a lower risk of severely depressed mood as compared to infrequent (<1x/wk.) intake, while no significant difference was observed when comparing frequent (>2x/wk.) and infrequent (<1x/wk.) legume intake [111]. The triple-blind RCT, conducted in post-menopausal women (*n* = 60), showed that daily supplementation with 30 mg of saffron for 6 weeks significantly reduced depressive symptoms compared to a placebo [106].

#### 3.2.3. Macronutrients

*Total Carbohydrate Intake*

The association between total carbohydrate intake and depressive symptoms was examined in 1 cross-sectional study (*n* = 1906) conducted in peri- and post-menopausal women [110]. The study found no significant association [110].

*Dietary Fiber Intake*

The association between dietary fiber intake and depressive symptoms was examined in 3 cross-sectional studies conducted in pre-, peri-, and post-menopausal women [81,96,110], 1 prospective cohort study conducted in post-menopausal women [88], and 1 RCT conducted in peri- and post-menopausal women [113]. Among the cross-sectional studies, 2 (*n* = 393 [96] and 6060 [81]) found no association between dietary fiber intake and depressive symptoms, whereas the other (*n* = 1906 [110]) found a negative association. The cohort study (*n* = 69,954; 3-year follow-up) also found a negative a negative association [88], while the 6-week RCT (*n* = 81), which compared prebiotic-enriched yogurt to regular yogurt, found no significant effect [113].

*Total Fat Intake*

The association between total fat intake and depressive symptoms was examined in 1 cross-sectional study (*n* = 1960) conducted in peri- and post-menopausal women [110]. The study found no significant association [110].

*MUFA Intake*

The association between MUFA intake and depressive symptoms was examined in 2 cross-sectional studies, 1 conducted in peri- and post-menopausal women [110], and 1 conducted in pre- and peri-menopausal women [78]. One (*n* = 1906) found no significant association [110], while the other (*n* = 3305) found positive associations between oleic and linoleic acid intakes and depressive symptoms, regardless of menopause status [78].

*PUFA Intake*

*Total PUFA Intake*: The association between total PUFA intake and depressive symptoms was examined in 1 cross-sectional study (*n* = 1960) conducted in peri- and post-menopausal women [110]. The study found no significant association [110].

*Total Omega-3 PUFA Intake*: The association between total omega-3 PUFA intake and depressive symptoms was examined in 2 cross-sectional studies [77,110] and a 3-year prospective cohort study [89]. One cross-sectional study (*n* = 1305) conducted in peri-menopausal women found a negative association [77], while the other (*n* = 1906) cross-sectional study conducted in peri- and post-menopausal women [110], as well as the cohort study (*n* = 1746; 3-year follow-up) conducted in post-menopausal women [89], reported no significant association.

*DHA Intake*: The association between DHA intake and depressive symptoms was examined in 2 prospective cohort studies, both conducted in post-menopausal women and spanning 3 years [89,104]. One study (*n* = 1746; 3-year follow-up) found no significant association [89], whereas the other (*n* = 2157; 3-year follow-up) found a positive association [104].

*EPA Intake*: The association between EPA intake and depressive symptoms was examined in 2 prospective cohort studies, both conducted in post-menopausal women and spanning 3 years [89,104]. One study (*n* = 1746; 3-year follow-up) found no significant association [89], whereas the other (*n* = 2157; 3-year follow-up) found a positive association [104].

*DHA+EPA Intake*: The association between DHA+EPA intake and depressive symptoms was examined in 2 prospective cohort studies conducted in post-menopausal women [89,104], 1 quasi-experimental pretest-post-test NEGD trial conducted in post-menopausal women [92], and 1 RCT conducted in peri- and post-menopausal women [112]. Findings from cohort studies were mixed: 1 study (*n* = 1746; 3-year follow-up) [89] found a negative association, while the other (*n* = 2157; 3-year follow-up) [104] found a positive association. In the 8-week pretest-post-test NEGD trial (*n* = 20), daily supplementation with DHA (375 mg) and EPA (465 mg) significantly reduced depressive symptoms from pre- to post-intervention [92]. Similarly, the 8-week RCT (*n* = 120) found that DHA (150 mg) + EPA (1005 mg) supplementation resulted in greater symptom reduction than placebo, but only in women without major depression at baseline (~75% of the sample) [112].

*Total Omega-6 PUFA Intake*: The association between total omega-6 PUFA intake and depressive symptoms was examined in 1 cross-sectional study (*n* = 1960) conducted in peri- and post-menopausal women [110]. The study found no significant association [110].

*SFA Intake*

The association between SFA intake and depressive symptoms was examined in 1 cross-sectional study conducted in post-menopausal women [110] and 1 prospective cohort study conducted in pre- and peri-menopausal women [85]. The cross-sectional study (*n* = 1906) found no significant association [110], whereas the cohort study (*n* = 1579; 4-year follow-up) found a positive association, regardless of menopause status [85].

*TFA Intake*

The association between TFA intake and depressive symptoms was examined in 1 cross-sectional study conducted in peri-menopausal women [79] and 1 prospective cohort study conducted in pre- and peri-menopausal women [86]. The cross-sectional study (*n* = 1416) found no significant association [79], whereas the cohort study (*n* = 3004; 5-year follow-up) found a positive association, regardless of menopause status [86].

*Total Protein Intake*

The association between total protein intake and depressive symptoms was examined in 1 cross-sectional study (*n* = 1960) conducted in peri- and post-menopausal women [110]. The study found no significant association [110].

#### 3.2.4. Micronutrients

*Vitamin A Precursors*

The association between plasma vitamin A levels, provitamin A intake, and β-carotene intakes and depressive symptoms were examined in 3 cross-sectional studies conducted in peri- and post-menopausal women [82,84,109]. None of the studies found significant associations [82,84,109].

*Vitamin C Intake*

The association between vitamin C intake and depressive symptoms was examined in 2 cross-sectional studies: 1 conducted in peri- and post-menopausal women (*n* = 1906) [110] and the other in pre- and peri-menopausal women (*n* = 3043) [84]. Both studies found negative associations [84,110].

*Vitamin D_3_ Intake or Status*

The association between vitamin D_3_ intake or status and depressive symptoms was examined in 2 cross-sectional studies conducted in peri- and post-menopausal women [110,114], 1 prospective cohort study conducted in post-menopausal women [87], and 2 RCTs conducted in post-menopausal women [90,107]. One cross-sectional study (*n* = 56) found no significant association between vitamin D_3_ intake and depressive symptoms [114], whereas the other (*n* = 1906) found a negative association [110]. Supporting the latter, the cohort study (*n* = 81,189; 3-year follow-up) found a negative association between vitamin D_3_ intake at study baseline and depressive symptoms at follow-up [87]. In contrast, 2 triple-blind RCTs, 1 testing daily supplementation with 1000 IU of vitamin D_3_ over 3 years (*n* = 36,282) [90] and the other testing 2000 IU daily for 1 year (*n* = 218) [107], found no significant effect on depressive symptoms compared with placebo.

*Vitamin E Intake or Plasma Levels*

The association between plasma vitamin E levels and depressive symptoms was examined in 1 cross-sectional study conducted in peri- and post-menopausal women (*n* = 1770), which found no significant association [109]. The effect of daily supplementation with 1000 mg of vitamin E for an 8-week period was also assessed in 1 triple-blind RCT conducted in post-menopausal women (*n* = 81), but no significant difference was observed when compared to placebo [105].

*Vitamin B_6_ Intake*

The association between vitamin B_6_ intake and depressive symptoms was examined in 1 cross-sectional study (*n* = 1960) conducted in peri- and post-menopausal women [110]. The study found no significant association [110].

*Vitamin B_9_ Intake or Plasma/Serum Levels*

The association between vitamin B_9_ intake and depressive symptoms was examined in 1 cross-sectional study conducted in peri- and post-menopausal women (*n* = 1906), which found no significant association [110]. The association between plasma vitamin B_9_ levels and depressive symptoms was also examined in 2 cross-sectional studies, 1 conducted in peri- and post-menopausal women (*n* = 1770) [109] and the other conducted in post-menopausal women (*n* = 96) [101], with no significant association observed.

*Vitamin B_12_ Intake or Plasma Levels*

The association between vitamin B_12_ intake and depressive symptoms was examined in 1 cross-sectional study conducted in peri- and post-menopausal women (*n* = 1906), which found a negative association [110]. The association between plasma vitamin B_12_ levels and depressive symptoms was also examined in 1 cross-sectional study conducted in post-menopausal women (*n* = 96), which found no significant association [101].

*Magnesium Intake or Plasma/Serum Levels*

The association between magnesium intake and depressive symptoms was examined in 1 cross-sectional study conducted in peri- and post-menopausal women (*n* = 1906), which found a negative association [110]. The association between plasma/serum magnesium levels and depressive symptoms was also examined in 2 cross-sectional studies [102,103] and 1 case-control study [99], all conducted in post-menopausal women. One cross-sectional study (*n* = 323) [102] and the case-control study (*n* = 171) [99] found a negative association, while the other cross-sectional study (*n* = 298) [103] found no significant association.

*Zinc Intake or Plasma/Serum Levels*

The association between zinc intake and depressive symptoms was examined in 1 cross-sectional study conducted in peri- and post-menopausal women (*n* = 1906), which found a negative association [110]. The association between plasma/serum zinc levels and depressive symptoms was also examined in 2 cross-sectional studies [102,103] and 1 case-control study [99], all conducted in post-menopausal women. One cross-sectional study (*n* = 323) [102] and the case-control study (*n* = 171) [99] found a negative association, while the other cross-sectional study (*n* = 298) [103] found no significant association.

*Selenium Intake or Plasma Levels*

The association between selenium intake and depressive symptoms was examined in 1 cross-sectional conducted in peri- and post-menopausal women (*n* = 1906), which found no significant association [110]. The association between plasma selenium levels and depressive symptoms was also examined in 1 cross-sectional study conducted in post-menopausal women (*n* = 96) [101], with no significant association observed.

*Copper Serum Levels*

The association between serum copper levels and depressive symptoms was assessed in 1 cross-sectional study (*n* = 298) involving post-menopausal women [103]. The study found no significant association [103].

*Manganese Intake*

The association between manganese intake and depressive symptoms was examined in 1 cross-sectional study (*n* = 1359) involving early peri-menopausal women [79]. The study found no significant association [79].

#### 3.2.5. Other Food Components

*Curcumin*

The effect of daily supplementation with 1000 mg of curcumin for an 8-week period on depressive symptoms was examined in 1 triple-blind RCT conducted in a sample of 81 post-menopausal women [105]. No significant difference was observed between the curcumin and the placebo groups [105].

## 4. Discussion

### 4.1. Summary of Study Findings

This scoping review identified and synthesized the currently available evidence on the association between diet and depression in peri- and post-menopausal women. A total of 38 studies were included, encompassing both observational and interventional designs. Most studies were either cross-sectional or cohort in nature and published within the past decade. These studies primarily explored the relationship between dietary patterns, nutrient intake, or specific foods and depressive symptoms. A smaller number of RCTs evaluated the effects of nutritional interventions.

A consistent pattern emerged across the literature: adherence to “healthy” dietary patterns, characterized by higher intakes of fruits, vegetables, whole grains, and lean proteins, was associated with a lower risk of depressive symptoms, while “unhealthy” dietary patterns, characterized by higher consumption of processed foods, added sugars, and saturated fats, were associated with a higher risk of depressive symptoms.

In contrast to dietary patterns, findings related to individual nutrients, food components, and specific foods or food groups were more heterogeneous and inconclusive. For example, studies examining specific omega-3 fatty acids, B vitamins, magnesium, zinc, and other micronutrients yielded mixed results, with many reporting a combination of positive, negative, and null associations with depressive symptoms. Similarly, studies on specific foods and food groups (e.g., fruit, vegetables, dairy) did not reveal any clear or consistent patterns of association with depressive symptoms.

### 4.2. Findings in Relation to Other Studies

The findings of this scoping review on peri- and post-menopausal women generally align with the broader nutritional psychiatry literature in adult populations. Numerous prior reviews and meta-analyses focused predominantly on the general adult population have shown diets rich in plant foods (vegetables, fruits, legumes, whole grains) and fish, often characterized as “Mediterranean”, “anti-inflammatory”, or other “healthy” patterns, are associated with a significantly lower risk of depression or depressive symptoms [29,30,31,32,33], whereas highly processed “Western-style” diets are associated with higher depression risk [34,35,36,37]. For instance, a recent systematic review and meta-analysis of RCTs reported that Mediterranean diet interventions significantly reduced depressive symptoms in young and middle-aged adults with major depression or mild-to-moderate depressive symptoms [32]. Similarly, an umbrella review reported significant inverse associations between adherence to Mediterranean and anti-inflammatory dietary patterns (reflected by low DII scores) and depression risk [115].

Our scoping review corroborates that these pattern-level associations are similarly observed in women undergoing the menopausal transition. This alignment is particularly noteworthy, as it suggests that the core associations between diet and depressive symptoms persist despite the distinct hormonal and metabolic changes characteristic of women’s midlife. The inconsistencies noted for individual foods and nutrients also mirror some of the findings reported in broader adult populations [116,117], and may, in part, be attributable to the limited ability of reductionist approaches to capture the complex interactions between nutrients and the overall dietary context.

Despite these similitudes, our review adds meaningful nuance to the existing literature, with our results also identifying patterns and inconsistencies that might be less visible in the overall adult population data. While some associations observed in general populations appear to hold true in peri- and post-menopausal women, others may manifest differently. For example, one cohort study reported that moderate legume intake was negatively associated with depressed mood in peri-menopausal women, but not in their post-menopausal counterparts [111]. Similarly, data from the SWAN study indicated that higher omega-3 fatty acid intake was associated with fewer depressive symptoms in peri-menopausal, but not pre-menopausal, women [77]. These discrepancies suggest that menopausal status may act as a modifier of the relationship between diet and depression.

Several biological mechanisms may be involved in these stage-specific differences, including hormonal fluctuations, a heightened proinflammatory milieu, and changes in gut microbiota composition. Estrogen is involved in the regulation of several physiological functions, including serotonergic and dopaminergic activity, the HPA axis, and neuroinflammation. During peri-menopause, fluctuating estrogen levels create a state of neuroendocrine instability that may heighten sensitivity to dietary influences [118]. This could explain why the SWAN study found protective effects of omega-3 intake in peri-menopausal but not pre-menopausal women [77,118], as the anti-inflammatory and membrane-stabilizing properties of omega-3 may be especially relevant during periods of hormonal volatility [119]. In contrast, once estrogen stabilizes at a chronically low level in post-menopause, such dietary effects may be attenuated, as seen in the null findings for legumes in post-menopausal women [111,118].

Gut microbiome alterations associated with menopause offer another explanatory pathway. Menopause is linked to reduced microbial diversity and diminished function of the estrobolome, the subset of gut microbes that regulates estrogen metabolism [120,121,122]. These microbial changes influence systemic estrogen availability and may alter how dietary exposures affect mood via the gut–brain axis [120,121,122]. Thus, it is plausible that fiber-rich legumes, which promote beneficial microbial metabolites such as short-chain fatty acids, could interact more strongly with fluctuating estrogen levels during peri-menopause to influence mood, whereas the blunted estrobolome activity in post-menopause could weaken this effect [120,121,122].

Taken together, these examples suggest that diet–depression associations may be potentiated or altered in the unique hormonal and microbial environment of peri-menopause but diminish or change once women enter post-menopause. While the number of studies specifically targeting peri- and post-menopausal women remains limited, our findings highlight the need for mechanistically informed, stage-specific dietary research in this demographic.

### 4.3. Strengths and Limitations of the Evidence

While some studies demonstrated methodological strengths, such as the use of validated instruments and appropriate statistical adjustments, the overall risk of bias in the evidence base was substantial. Over 70% of observational studies were rated as high risk of bias, often due to selection bias, residual confounding, or poor handling of dietary exposures and depression measures. These limitations significantly reduce confidence in many reported associations, particularly for exposures with limited replication or inconsistent findings.

Most included studies were observational, inherently limiting causal inference. Among the relatively few RCTs, many employed open-label designs without active comparators, reducing internal validity. Definitions of peri- and post-menopause varied, with some studies relying solely on age-based thresholds rather than standardized clinical staging criteria, thereby increasing the risk of misclassification. Dietary exposures were predominantly assessed using self-reported methods, such as food frequency questionnaires or 24 h recalls, which are known to be prone to recall bias and measurement error. Similarly, depressive symptoms were measured using a range of instruments, contributing to outcome heterogeneity. Residual confounding remains a critical concern, particularly in relation to unmeasured or insufficiently adjusted variables such as physical activity, severity of menopausal symptoms, and psychosocial stressors. These challenges are especially pertinent to the nutrition research field, where exposures are complex, error-prone, and strongly interrelated with other lifestyle and behavioral factors.

### 4.4. Strengths and Limitations of the Review

To date, this review represents the most comprehensive synthesis on diet and depression in peri- and post-menopausal women. It was conducted using rigorous methodology, including prospective protocol registration, comprehensive database searches, and dual independent screening. Additionally, while not a mandatory step in the scoping review methodology, a formal risk of bias assessment of the included studies was conducted, enhancing the ability to identify key methodological limitations and have an overall portrait of the quality of the evidence. Adherence to the PRISMA-ScR reporting guidelines further strengthened the methodological transparency, reproducibility, and credibility of the review process.

However, certain limitations also warrant consideration. The inclusion of only English- and French-language publications may have introduced language bias and excluded relevant studies published in other languages. Additionally, due to the wide variability in populations and dietary exposures across studies, our synthesis was necessarily descriptive rather than quantitative.

### 4.5. Research Gaps and Future Directions

This scoping review highlights several critical gaps in the current literature that warrant further investigation:

- Limited interventional research: Few RCTs have specifically examined dietary interventions in peri- and post-menopausal women. Future RCTs are needed to evaluate the effectiveness of dietary strategies for preventing or managing depressive symptoms during and after the menopausal transition.
- Lack of standardization in exposure and outcome measures: Greater consistency is needed in how dietary exposures (e.g., specific dietary patterns, nutrient intake) and depressive outcomes (e.g., validated symptom scales, diagnostic criteria) are defined and measured. Standardization would improve the comparability and synthesis of findings across studies.
- Inadequate consideration of menopause-specific factors: Many studies overlook important contextual variables such as hormonal status, severity of menopausal symptoms, and hormone therapy use. These factors may act as effect modifiers and should be systematically considered in future research to better understand the diet–depression relationship in this population.
- Limited population diversity: Existing studies are often restricted to specific geographic regions or demographically homogeneous cohorts. There is a need for research in more diverse populations to capture how cultural, socioeconomic, and racial/ethnic factors influence dietary intakes and mental health outcomes in midlife women.
- Insufficient investigation of biological mechanisms: Although mechanisms such as sub-chronic inflammation, oxidative stress, and gut microbiota dysbiosis have been proposed, few studies have directly assessed these pathways in peri- and post-menopausal women. Incorporating biomarker analyses and mechanistic approaches into future studies could provide critical insights into how diet influences mental health during midlife.

### 4.6. Implications for Research and Practice

As the burden of depression continues to rise in midlife women, and given the limitations of current pharmacological treatments, there is a crucial need to explore effective non-pharmacological strategies. Dietary modifications represent a promising avenue, with preliminary evidence suggesting that healthy dietary patterns may help protect against depression during the peri- and post-menopausal periods. However, the current evidence base remains limited by its predominantly observational design, heterogeneity in dietary assessment methods, variability in outcome measures, and a lack of standardized definitions of menopausal status. Longitudinal studies with rigorous methodologies and high-quality RCTs are crucially needed to establish causal relationships, clarify underlying biological mechanisms, and develop specific, evidence-based dietary recommendations for this population.

When interpreting dietary associations, clinicians and researchers should carefully consider the underlying methodological quality. Observational findings, particularly those derived from studies at high risk of bias, should be viewed as hypothesis-generating rather than conclusive. Well-powered, methodologically rigorous trials remain essential for informing clinical recommendations. In practice, healthcare providers working with peri- and post-menopausal women could consider diet as one component of a broader, integrative approach to mental health promotion. While acknowledging the limitations of the existing evidence, encouraging dietary patterns known to benefit physical health may also provide ancillary mental health benefits. Similarly, public health initiatives aimed at promoting healthy eating habits in midlife women could yield the dual advantage of improving both mental and cardiometabolic health outcomes, thereby simultaneously addressing multiple health priorities.

## 5. Conclusions

This scoping review offers the most comprehensive synthesis to date of the relationship between diet and depression in peri- and post-menopausal women. The most consistent evidence supports a negative association between healthy dietary patterns and depressive symptoms, and a positive association between unhealthy dietary patterns and depression. Findings for individual nutrients were more variable, with vitamin D_3_ and dietary fiber showing the most promise, despite some inconsistencies.

In addition to the limited volume of studies available on each dietary exposure, the overall poor methodological quality of included studies limits the certainty of findings presented in this review. As such, these results should inform future hypothesis generation and study design rather than immediate clinical recommendations. To advance the field, future research should prioritize high-quality study designs, standardized definitions of menopausal status and depression assessments, and targeted investigations of key exposures identified here. While tailored dietary interventions may hold promise for the prevention and management of depression and depressive symptoms in peri and post-menopausal women, robust causal evidence is still needed.

## Data Availability

All available data is provided as Supplementary Materials.

## Supplementary Materials

The following supporting information can be downloaded at: https://www.mdpi.com/article/10.3390/nu17172846/s1, Table S1: Preferred Reporting Items for Systematic Reviews and Meta-Analyses Checklist’s Extension for Scoping Review (PRISMA-ScR); Table S2: Full search strategy for Medline; Table S3: Full search strategy for Embase; Table S4: full search strategy for PsycInfo; Table S5: Full search strategy for CENTRAL; Table S6: Full search strategy for Web of Science; Table S7: Full search strategy for Scopus; Table S8: Papers excluded after full-text review; Table S9: Risk of bias assessment for cross-sectional (*n* = 22) and prospective cohort (*n* = 7) studies; Table S10: Risk of bias assessment for case-control studies (*n* = 1); Table S11: Risk of bias assessment for experimental studies (*n* = 9).

## Abbreviations

The following abbreviations are used in this manuscript:

BDI	Beck’s Depression Inventory
BS	Burnam Scale
BSI	Brief Symptom Inventory
CENTRAL	Cochrane Central Register of Controlled Trials
CES-D	Centre for Epidemiologic Studies Depression Scale
DASH	Dietary Approach to Stop Hypertension
DASS	Depression Anxiety Stress Scale
DHA	Docosahexaenoic Acid
DII	Dietary Inflammatory Index
DTAC	Dietary Total Antioxidant Capacity
EPA	Eicosapentaenoic Acid
GCS	Greene Climacteric Scale
HDRS	Hamilton Depression Rating Scale
HPA	Hypothalamic–pituitary–adrenal
HSCL-D	Hopkins Symptoms Checklist Depression Scale
MADRS	Montgomery–Åsberg Depression Rating Scale
MUFA(s)	Monounsaturated Fatty Acid(s)
NEGD	Non-Equivalent Groups Design
NHLBI	National Heart, Lung, and Blood Institute
PHQ-9	Patient Health Questionnaire
POMS	Profile of Mood State
PRISMA	Preferred Reporting Items for Systematic Reviews Meta-Analyses
PRISMA-ScR	PRISMA Checklist’s Extension for Scoping Reviews
PUFA(s)	Polyunsaturated Fatty Acid(s)
RCT(s)	Randomized controlled trial(s)
RoB	Risk of Bias
SFA(s)	Saturated Fatty Acid(s)
SWAN	Studies of Women’s Health Across the Nation
TFA(s)	Trans Fatty Acid(s)
WHI	Women’s Health Initiative
WHQ	Women’s Health Questionnaire
ZSRDS	Zung Self-Rating Depression Scale
