# Supplementary material for: Diet and Depression During Peri- and Post-Menopause: A Scoping Review
Source: Nutrients. 2025 Aug 31;17(17):2846. doi: 10.3390/nu17172846 (PMC12429875; doi:10.3390/nu17172846)
Supplement: Supplementary file 1 [file nutrients-17-02846-s001.zip › nutrients-3772327-supplementary.pdf]

SUPPLEMENTARY MATERIALS

TABLE OF CONTENTS

SUPPLEMENTARY MATERIALS 1

- Table S1. PRISMA Checklist Extension for Scoping Review (PRISMA-ScR) 2
- Table S2. Full Search Strategy for Medline 3
- Table S3. Full Search Strategy for Embase 4
- Table S4. Full Search Strategy for PsycINFO 5
- Table S5. Full Search Strategy for CENTRAL 6
- Table S6. Full Search Strategy for Web of Science 7
- Table S7. Full Search Strategy for Scopus 8
- Table S8. Papers excluded after full text review 9
- Table S9. Risk of Bias Assessment for Cross-Sectional (n=22) and Prospective Cohort (n=7) Studies 10
- Table S10. Risk of Bias Assessment for Case-Control Studies (n=1) 11
- Table S11. Risk of Bias Assessment for Experimental Studies (n=9) 12

Table S1. PRISMA Checklist Extension for Scoping Review (PRISMA-ScR)

SECTION	ITEM	PRISMA-ScR CHECKLIST ITEM	PAGE #
**TITLE**			
Title	1	Identify the report as a scoping review.	1
**ABSTRACT**			
Structured summary	2	Provide a structured summary that includes (as applicable): background, objectives, eligibility criteria, sources of evidence, charting methods, results, and conclusions that relate to the review questions and objectives.	1
**INTRODUCTION**			
Rationale	3	Describe the rationale for the review in the context of what is already known. Explain why the review questions/objectives lend themselves to a scoping review approach.	1-3
Objectives	4	Provide an explicit statement of the questions and objectives being addressed with reference to their key elements (e.g., population or participants, concepts, and context) or other relevant key elements used to conceptualize the review questions and/or objectives.	3
**METHODS**			
Protocol and registration	5	Indicate whether a review protocol exists; state if and where it can be accessed (e.g., a Web address); and if available, provide registration information, including the registration number.	3
Eligibility criteria	6	Specify characteristics of the sources of evidence used as eligibility criteria (e.g., years considered, language, and publication status), and provide a rationale.	3-4
Information sources	7	Describe all information sources in the search (e.g., databases with dates of coverage and contact with authors to identify additional sources), as well as the date the most recent search was executed.	3
Search	8	Present the full electronic search strategy for at least 1 database, including any limits used, such that it could be repeated.	3
Selection of sources of evidence	9	State the process for selecting sources of evidence (i.e., screening and eligibility) included in the scoping review.	4
Data charting process	10	Describe the methods of charting data from the included sources of evidence (e.g., calibrated forms or forms that have been tested by the team before their use, and whether data charting was done independently or in duplicate) and any processes for obtaining and confirming data from investigators.	4-5
Data items	11	List and define all variables for which data were sought and any assumptions and simplifications made.	4-5
Critical appraisal of individual sources of evidence	12	If done, provide a rationale for conducting a critical appraisal of included sources of evidence; describe the methods used and how this information was used in any data synthesis (if appropriate).	5
Synthesis of results	13	Describe the methods of handling and summarizing the data that were charted.	5
**RESULTS**			
Selection of sources of evidence	14	Give numbers of sources of evidence screened, assessed for eligibility, and included in the review, with reasons for exclusions at each stage, ideally using a flow diagram.	5-6
Characteristics of sources of evidence	15	For each source of evidence, present characteristics for which data were charted and provide the citations.	6-7
Critical appraisal within sources of evidence	16	If done, present data on critical appraisal of included sources of evidence (see item 12).	7
Results of individual sources of evidence	17	For each included source of evidence, present the relevant data that were charted that relate to the review questions and objectives.	8-18
Synthesis of results	18	Summarize and/or present the charting results as they relate to the review questions and objectives.	8-18
**DISCUSSION**			
Summary of evidence	19	Summarize the main results (including an overview of concepts, themes, and types of evidence available), link to the review questions and objectives, and consider the relevance to key groups.	18
Limitations	20	Discuss the limitations of the scoping review process.	19-20
Conclusions	21	Provide a general interpretation of the results with respect to the review questions and objectives, as well as potential implications and/or next steps.	20-21
**FUNDING**			
Funding	22	Describe sources of funding for the included sources of evidence, as well as sources of funding for the scoping review. Describe the role of the funders of the scoping review.	21

Table S2. Full Search Strategy for Medline

Database: MEDLINE(R) ALL 1946 to November 14, 2024
Platform: Ovid
Date searched: November 15, 2024

#	Search terms	Results
1	exp diet/ or exp nutritive value/ or exp hunger/	372,131
2	nutritional physiological phenomena/ or eating/ or feeding behavior/ or appetite regulation/	178,235
3	food/ or dairy products/ or dietary carbohydrates/ or dietary fats/ or dietary fiber/ or dietary proteins/ or dietary supplements/ or eggs/ or fast foods/ or fruit/ or meals/ or meat/ or micronutrients/ or nuts/ or seeds/ or vegetables/	412,074
4	diet*.ti,ab,kw.	737,299
5	nutriti*.ti,ab,kw.	408,207
6	food.ti,ab,kw.	603,814
7	eat*.ti,ab,kw.	136,072
8	energy intake.ti,ab,kw.	25,796
9	(macronutrient* or micronutrient* or nutrient*).ti,ab,kw.	235,467
10	or/1-9	1,981,681
11	exp depressive disorder/	126,528
12	depression/	163,505
13	mood disorders/	16,422
14	(depression* or (depressive adj3 (condition* or disorder* or symptom*))).ti,ab,kw.	512,225
15	or/11-14	574,799
16	exp menopause/	65,073
17	(menopaus* or perimenopaus* or postmenopaus*).ti,ab,kw.	110,372
18	or/16-17	125,265
19	10 and 15 and 18	406
20	limit 19 to (english or french)	389
21	limit 20 to humans	305
22	limit 21 to female	290

Table S3. Full Search Strategy for Embase

Database: Embase Classic+Embase 1947 to November 14, 2024
Platform: Ovid
Date searched: November 15, 2024

#	Search terms	Results
1	exp diet/ or exp food intake/ or exp feeding behavior/	1,044,379
2	food/ or dairy product/ or dietary fiber/ or dietary supplement/ or egg/ or fast food/ or fat/ or fruit/ or health food/ or meat/ or nut/ or sugar/ or vegetable/	432,386
3	diet*.ti,ab,kw.	991,865
4	nutriti*.ti,ab,kw.	549,610
5	food.ti,ab,kw.	743,805
6	eat*.ti,ab,kw.	187,438
7	energy intake.ti,ab,kw.	34,297
8	(macronutrient* or micronutrient* or nutrient*).ti,ab,kw.	278,794
9	or/1-8	2,701,257
10	depression/	535,086
11	mood disorder/	57,176
12	(depression* or (depressive adj3 (condition* or disorder* or symptom*))).ti,ab,kw.	700,670
13	or/10-12	900,176
14	exp "menopause and climacterium"/	148,545
15	menopause/	57,165
16	(menopaus* or perimenopaus* or postmenopaus*).ti,ab,kw.	164,326
17	or/14-16	204,563
18	9 and 13 and 17	1,003
19	limit 18 to (english or french)	961
20	limit 19 to humans	887
21	limit 20 to female	674

Table S4. Full Search Strategy for PsycINFO

Database: APA PsycInfo 1806 to November 2024 Week 2
Platform: Ovid
Date searched: November 15, 2024

#	Search terms	Results
1	exp appetite/	6,749
2	diets/ or food intake/ or eating behavior/	44,851
3	diet*.ti,ab,kw.	63,713
4	nutriti*.ti,ab,kw.	41,612
5	food.ti,ab,kw.	109,112
6	eat*.ti,ab,kw.	85,986
7	energy intake.ti,ab,kw.	5,978
8	(macronutrient* or micronutrient* or nutrient*).ti,ab,kw.	6,853
9	or/1-8	219,046
10	major depression/	164,494
11	dysthymic disorder/	1,544
12	recurrent depression/	1,060
13	treatment resistant depression/	3,413
14	(depression* or (depressive adj3 (condition* or disorder* or symptom*))).mp.	435,053
15	or/10-14	435,255
16	Menopause/	4,359
17	(menopaus* or perimenopaus* or postmenopaus*).mp.	8,054
18	or/16-17	8,054
19	9 and 15 and 18	172
20	limit 19 to (english or french)	168
21	limit 20 to female	141

Table S5. Full Search Strategy for CENTRAL

Database: EBM Reviews – Cochrane Central Register of Controlled Trials October 2024
Platform: Ovid
Date searched: November 15, 2024

#	Search terms	Results
1	diet*.ti,ab,kw.	121,776
2	nutriti*.ti,ab,kw.	59,762
3	food.ti,ab,kw.	62,001
4	eat*.ti,ab,kw.	25,932
5	energy intake.ti,ab,kw.	10,179
6	(macronutrient* or micronutrient* or nutrient*).ti,ab,kw.	15,353
7	or/1-6	195,966
8	(depression* or (depressive adj3 (condition* or disorder* or symptom*))).mp.	114,947
9	(menopaus* or perimenopaus* or postmenopaus*).mp.	32,912
10	7 and 8 and 9	206

Table S6. Full Search Strategy for Web of Science

Database: Web of Science 1953 to November 2024
Platform: Web of Science
Date searched: November 15, 2024

#	Search terms	Results
1	((TS=(nutrient* OR micronutrient* OR macronutrient* OR "energy intake" OR eat* OR food OR nutriti* OR diet*)))	3,105,057
2	(((TS=(depression* OR "depression condition*" OR "depression disorder*" OR "depression symptom*"))))	741,572
3	(((TS=(menopaus* OR perimenopaus* OR postmenopaus*))))	159,125
4	#3 AND #2 AND #1	469
5	#4 AND LA=(English OR French)	456
6	#5 AND TS=(Female)	111

Table S7. Full Search Strategy for Scopus

Database: Scopus Before 1960 to November 2024
Platform: Scopus
Date searched: November 15, 2024

#	Search terms	Results
1	TITLE-ABS-KEY (nutrient*) OR TITLE-ABS-KEY (micronutrient*) OR TITLE-ABS-KEY (macronutrient*) OR TITLE-ABS-KEY ("energy intake") OR TITLE-ABS-KEY (eat*) OR TITLE-ABS-KEY (food) OR TITLE-ABS-KEY (nutriti*) OR TITLE-ABS-KEY (diet*)	4,090,157
2	TITLE-ABS-KEY (depression*) OR TITLE-ABS-KEY ("depression condition*") OR TITLE-ABS-KEY ("depression disorder*") OR TITLE-ABS-KEY ("depression symptom*")	968,397
3	TITLE-ABS-KEY (menopaus*) OR TITLE-ABS-KEY (perimenopaus*) OR TITLE-ABS-KEY (postmenopaus*)	183,748
4	#1 AND #2 AND #3	1,101
5	#4 AND (LIMIT-TO (LANGUAGE, "English") OR LIMIT-TO (LANGUAGE, "French"))	1,032
6	#5 AND LIMIT-TO (EXACTKEYWORD, "Human")	955

Table S8. Papers excluded after full text review

Reason for exclusion: Ineligible language (i.e., language other than English and French) n=2

1. Jokar, A.; Farahi, F. Effect of Vitamin C on Depression in Menopausal Women with Balanced Diet: A Randomized Clinical Trial. The Iranian Journal of Obstetrics, Gynecology and Infertility 2014, 17, 18–23, doi:10.22038/ijogi.2014.3557.
2. Shahraeini, M.; Shourab, N.J.; Javan, R.; Shakeri, M.T. Effect of Food-Based Strategies of Iranian Traditional Medicine on Women’s Quality of Life during Menopause. The Iranian Journal of Obstetrics, Gynecology and Infertility 2021, 23, 67–75, doi:10.22038/ijogi.2021.17828.

Reason for exclusion: Ineligible publication (i.e., one paper was a letter, one was reporting a literature review) n=2

1. Chocano-Bedoya, P.; O’Reilly, E.; Lucas, M.; Mirzaei, F.; Okereke, O.; Fung, T.; Hu, F.; Ascherio, A. Dietary Patterns and Depression in the Nurses’ Health Study. Am J Epidemiol 2012, 175, S75, doi:10.1093/aje/kws258.
2. Pasco, J.A.; Williams, L.J.; Brennan-Olsen, S.L.; Berk, M.; Jacka, F.N. Milk Consumption and the Risk for Incident Major Depressive Disorder. Psychother Psychosom 2015, 84, 384–386, doi:10.1159/000381831.

Reason for exclusion: Ineligible population (i.e., mixed menopausal stage population, without stratified analyses) n=7

1. Crawford, G.B.; Khedkar, A.; Flaws, J.A.; Sorkin, J.D.; Gallicchio, L. Depressive Symptoms and Self-Reported Fast-Food Intake in Midlife Women. Prev Med (Baltim) 2011, 52, 254–257, doi:10.1016/j.ypmed.2011.01.006.
2. Bae, Y.J.; Kim, S.K. Low Dietary Calcium Is Associated with Self-Rated Depression in Middle-Aged Korean Women. Nutr Res Pract 2012, 6, 527–533, doi:10.4162/nrp.2012.6.6.527.
3. Shivappa, N.; Schoenaker, D.A.J.M.; Hebert, J.R.; Mishra, G.D. Association between Inflammatory Potential of Diet and Risk of Depression in Middle-Aged Women: The Australian Longitudinal Study on Women’s Health. British Journal of Nutrition 2016, 116, 1077–1086, doi:10.1017/s0007114516002853.
4. Odai, T.; Terauchi, M.; Suzuki, R.; Kato, K.; Hirose, A.; Miyasaka, N. Depressive Symptoms in Middle-Aged and Elderly Women Are Associated with a Low Intake of Vitamin B6: A Cross-Sectional Study. Nutrients 2020, 12, 3437, doi:10.3390/nu12113437.
5. Park, S.J.; Lee, D.K.; Kim, B.; Na, K.S.; Lee, C.H.; Son, Y.D.; Lee, H.J. The Association between Omega-3 Fatty Acid Intake and Human Brain Connectivity in Middle-Aged Depressed Women. Nutrients 2020, 12, 2191, doi:10.3390/nu12082191.
6. Shon, J.; Seong, Y.; Choi, Y.; Kim, Y.; Cho, M.S.; Ha, E.; Kwon, O.; Kim, Y.; Park, Y.J.; Kim, Y. Meal-Based Intervention on Health Promotion in Middle-Aged Women: A Pilot Study. Nutrients 2023, Vol. 15, Page 2108 2023, 15, 2108, doi:10.3390/nu15092108.
7. Choi, J.Y.; Park, S.J.; Lee, H.J. Healthy and Unhealthy Dietary Patterns of Depressive Symptoms in Middle-Aged Women. Nutrients 2024, 16, 776, doi:10.3390/nu16060776.

Reason for exclusion: Ineligible intervention (i.e., were non-dietary components, phytoestrogens, and herbal extracts) n=4

1. Carels, R.A.; Darby, L.A.; Cacciapaglia, H.M.; Douglass, O.M. Reducing Cardiovascular Risk Factors in Postmenopausal Women through a Lifestyle Change Intervention. J Womens Health 2004, 13, 412–426, doi:10.1089/154099904323087105.
2. Palacios, S.; Mustata, C.; Rizo, J.M.; Regidor, P.A. Improvement in Menopausal Symptoms with a Nutritional Product Containing Evening Primrose Oil, Hop Extract, Saffron, Tryptophan, Vitamins B6, D3, K2, B12, and B9. European Review of Medical and Pharmacological Sciences 2023, 27, 8180–8189, doi:10.26355/eurrev_202309_33578.
3. Kachko, V.A.; Shulman, L.P.; Kuznetsova, I. V.; Uspenskaya, Y.B.; Burchakov, D.I. Clinical Evaluation of Effectiveness and Safety of Combined Use of Dietary Supplements Amberen® and Smart B® in Women with Climacteric Syndrome in Perimenopause. Adv Ther 2024, 41, 3183–3195, doi:10.1007/s12325-024-02910-0.
4. Liu, X.; Li, J.; He, D.; Zhang, D.; Liu, X. Association between Different Triglyceride Glucose Index-Related Indicators and Depression in Premenopausal and Postmenopausal Women: NHANES, 2013–2016. J Affect Disord 2024, 360, 297–304, doi:10.1016/j.jad.2024.05.084.

Reason for exclusion: Ineligible outcome (i.e., outcome of interest was not depression or depressive symptoms) n=3

1. Abshirini, M., Siassi, F., Koohdani, F., Qorbani, M., Khosravi, S., Hedayati, M., Aslani, Z., Soleymani, M., & Sotoudeh, G. (2018). Dietary total antioxidant capacity is inversely related to menopausal symptoms: a cross-sectional study among Iranian postmenopausal women. Nutrition, 55–56, 161–167. https://doi.org/10.1016/j.nut.2018.04.014
2. Shafie, M.; Homayouni Rad, A.; Mirghafourvand, M. Effects of Prebiotic-Rich Yogurt on Menopausal Symptoms and Metabolic Indices in Menopausal Women: A Triple-Blind Randomised Controlled Trial. Int J Food Sci Nutr 2022, 73, 693–704, doi:10.1080/09637486.2022.2048360.
3. Haghshenas, N.; Baharanchi, F.H.; Melekoglu, E.; Sohouli, M.H.; Shidfar, F. Comparison of Predictive Effect of the Dietary Inflammatory Index and Empirically Derived Food-Based Dietary Inflammatory Index on the Menopause-Specific Quality of Life and Its Complications. BMC Womens Health 2023, 23, 1–13, doi:10.1186/s12905-023-02485-y

Table S9. Risk of Bias Assessment for Cross-Sectional (n=21) and Prospective Cohort (n=7) Studies

Study	Item 1	Item 2	Item 3	Item 4	Item 5	Item 6	Item 7	Item 8	Item 9	Item 10	Item 11	Item 12	Item 13	Item 14	Item 15	Total (/15)
Abshirini (2019a)*	1	1	NR	1	0	0	0	1	1	0	1	1	0	1	1	**9**
Azarmanesh (2022)*	1	1	NR	1	0	0	0	1	1	0	1	1	0	1	1	**9**
Bertone-Johnson (2021)**	1	1	NR	1	1	1	1	1	1	0	1	1	1	1	1	**13**
Chae (2021)*	1	1	NR	1	0	0	0	1	1	1	0	1	0	1	1	**9**
Colangelo (2017)**	1	1	NR	1	0	1	1	1	1	0	1	1	1	1	1	**12**
Gangwisch (2015)**	1	1	NR	1	1	1	1	1	1	0	1	1	1	1	1	**13**
Kim (2021)*	1	1	NR	1	1	0	0	1	1	0	1	1	0	1	1	**10**
Kostecka (2022)*	1	1	NR	1	0	0	0	1	0	0	1	1	0	1	1	**8**
Lee (2023)*	1	1	NR	1	0	0	0	1	1	0		1	0	1	1	**9**
Li (2010)**	1	1	NR	1	1	1	1	1	1	0	1	1	0	1	1	**12**
Li (2020a)*	1	1	NR	1	0	0	0	1	1	0	1	1	0	1	1	**9**
Li (2020b)**	1	1	NR	1	0	1	1	1	1	0	1	1	1	1	1	**12**
Li (2020c)*	1	1	NR	1	0	0	0	1	1	0	1	1	0	1	1	**9**
Li (2020d)*	1	1	NR	1	0	0	0	1	1	0	1	1	0	1	1	**9**
Li (2020e)**	1	1	NR	1	0	1	1	1	1	0	1	1	1	1	1	**12**
Li (2020f)*	1	1	NR	1	0	0	0	1	1	0	1	1	0	1	1	**9**
Li (2020g)*	1	1	NR	1	0	0	0	1	1	0	1	1	0	1	1	**9**
Li (2021)*	1	1	NR	1	0	0	0	1	1	0	1	1	0	1	1	**9**
Li (2022a)*	1	1	NR	1	0	0	0	1	1	0	1	1	0	1	1	**9**
Li (2022b)*	1	1	NR	1	0	0	0	1	1	0	1	1	0	1	1	**9**
Liao (2019)*	1	1	NR	1	0	0	0	1	1	0	1	1	0	1	1	**9**
Liu (2016)*	1	1	NR	1	0	0	0	1	1	0	1	1	0	1	1	**9**
Noll (2022)*	1	1	NR	1	0	0	0	1	1	0	1	1	0	1	1	**9**
Oldra (2020)*	1	1	NR	1	0	0	0	1	1	0	1	1	0	1	1	**9**
Persons (2014)**	1	1	NR	1	1	1	1	1	1	0	1	1	1	1	1	**13**
Sengul (2014)*	1	1	NR	1	0	0	0	0	1	0	1	1	0	0	1	**7**
Stanislawska (2014)*	1	1	NR	1	0	0	0	0	1	0	1	1	0	0	1	**7**
Wieder-Huszla (2020)*	1	1	NR	1	0	0	0	1	1	0	1	1	0	0	0	**7**

Items of the NHLBI Tool for Cross-Sectional and Cohort Studies
Item 1. Was the research question or objective in this paper clearly stated and appropriate?
Item 2. Was the study population clearly specified and defined?
Item 3. Was the participation rate of eligible persons at least 50%?
Item 4. Were all the subjects selected or recruited from the same or similar populations (including the same time period)? Were inclusion and exclusion criteria for being in the study prespecified and applied uniformly to all participants?
Item 5. Was a sample size justification, power description, or variance and effect estimates provided?
Item 6. For the analyses in this paper, were the exposure(s) of interest measured prior to the outcome(s) being measured?
Item 7. Was the timeframe sufficient so that one could reasonably expect to see an association between exposure and outcome if it existed?
Item 8. For exposures that can vary in amount or level, did the study examine different levels of the exposure as related to the outcome (e.g., categories of exposure, or exposure measured as continuous variable)?
Item 9. Were the exposure measures (independent variables) clearly defined, valid, reliable, and implemented consistently across all study participants?
Item 10. Was the exposure(s) assessed more than once over time?
Item 11. Were the outcome measures (dependent variables) clearly defined, valid, reliable, and implemented consistently across all study participants?
Item 12. Were the outcome assessors blinded to the exposure status of participants?
Item 13. Was loss to follow-up after baseline 20% or less?
Item 14. Were key potential confounding variables measured and adjusted statistically for their impact on the relationship between exposure(s) and outcome(s)?
Item 15. Did the authors report any potential sources of conflict of interest, including any funding they received for conducting the study?

NR: Not reported

Table S10. Risk of Bias Assessment for Case-Control Studies (n=1)

Study	Item 1	Item 2	Item 3	Item 4	Item 5	Item 6	Item 7	Item 8	Item 9	Item 10	Item 11	Item 12	Item 13	Total (/13)
Nazari (2019)	1	1	0	1	1	1	NR	0	0	1	1	0	0	**7**

Items of the NHLBI Tool for Case-Control Studies
Item 1. Was the research question or objective in this paper clearly stated and appropriate?
Item 2. Was the study population clearly specified and defined?
Item 3. Did the authors include a sample size justification?
Item 4. Were controls selected or recruited from the same or similar population that gave rise to the cases (including the same timeframe)?
Item 5. Were the definitions, inclusion and exclusion criteria, algorithms or processes used to identify or select cases and controls valid, reliable, and implemented consistently across all study participants?
Item 6. Were the cases clearly defined and differentiated from controls?
Item 7. If less than 100 percent of eligible cases and/or controls were selected for the study, were the cases and/or controls randomly selected from those eligible?
Item 8. Was there use of concurrent controls?
Item 9. Were the investigators able to confirm that the exposure/risk occurred prior to the development of the condition or event that defined a participant as a case?
Item 10. Were the measures of exposure/risk clearly defined, valid, reliable, and implemented consistently (including the same time period) across all study participants?
Item 11. Were the assessors of exposure/risk blinded to the case or control status of participants?
Item 12. Were key potential confounding variables measured and adjusted statistically in the analyses? If matching was used, did the investigators account for matching during study analysis?
Item 13. Did the authors report any potential sources of conflict of interest, including any funding they received for conducting the study?

NR: Not reported

Table S11. Risk of Bias Assessment for Experimental Studies (n=9)

**Study**	**Domain 1**	**Domain 2**	**Domain 3**	**Domain 4**	**Domain 5**	**Domain 6**	**Overall**
Assaf (2016)	Low	High	High	Low	Low	Low	**High**
Bertone-Johnson (2011)	Low	Low	High	Low	Low	Low	**High**
Farshbaf-Khalili (2022)	Low	Low	Low	Low	Low	Low	**Low**
Freeman (2011)	NA	High	High	Low	Low	Low	**High**
Kashani (2018)	Low	Low	Low	Low	Low	Low	**Low**
Lucas (2009)	Low	Low	Low	Low	Low	Low	**Low**
Mason (2016)	Low	Low	Low	Low	Low	Low	**Low**
Shafie (2022)	Low	Low	Low	Low	Low	Low	**Low**
Torres (2012)	Low	High	High	Low	Low	Low	**High**
**RoB-2 Items**<br>Domain 1 – Bias arising from the randomization process<br>Domain 2 – Bias arising from deviations from the intended intervention (effect of assignment to an intervention)<br>Domain 3 – Bias arising from deviations from the intended intervention (effect of adherence to an intervention)<br>Domain 4 – Bias arising from missing outcome data<br>Domain 5 – Bias arising from bias in the measurement of the outcome<br>Domain 6 – Bias arising from the selection of reported results							

NA: Not applicable
